# Structure and function of a spectrin-like regulator of bacterial cytokinesis

**DOI:** 10.1038/ncomms6421

**Published:** 2014-11-18

**Authors:** Robert M. Cleverley, Jeffrey R. Barrett, Arnaud Baslé, Nhat Khai Bui, Lorraine Hewitt, Alexandra Solovyova, Zhi-Qiang Xu, Richard A. Daniel, Nicholas E. Dixon, Elizabeth J. Harry, Aaron J. Oakley, Waldemar Vollmer, Richard J. Lewis

**Affiliations:** 1 Institute for Cell and Molecular Biosciences, Newcastle University, Newcastle upon Tyne NE2 4HH, UK; 2 Centre for Medical and Molecular Bioscience, University of Wollongong, Wollongong, New South Wales 2522, Australia; 3 NUPPA, Devonshire Building, Newcastle University, Newcastle upon Tyne NE1 7RU, UK; 4 The ithree Institute, University of Technology, Sydney, New South Wales 2007, Australia

## Abstract

Bacterial cell division is facilitated by a molecular machine—the divisome—that assembles at mid-cell in dividing cells. The formation of the cytokinetic Z-ring by the tubulin homologue FtsZ is regulated by several factors, including the divisome component EzrA. Here we describe the structure of the 60-kDa cytoplasmic domain of EzrA, which comprises five linear repeats of an unusual triple helical bundle. The EzrA structure is bent into a semicircle, providing the protein with the potential to interact at both N- and C-termini with adjacent membrane-bound divisome components. We also identify at least two binding sites for FtsZ on EzrA and map regions of EzrA that are responsible for regulating FtsZ assembly. The individual repeats, and their linear organization, are homologous to the spectrin proteins that connect actin filaments to the membrane in eukaryotes, and we thus propose that EzrA is the founding member of the bacterial spectrin family.

## Abstract (web summary)

EzrA regulates the polymerization of FtsZ, a tubulin-like protein and main component of the Z-ring, which drives cell division in bacteria. Here the authors describe the crystal structure of EzrA and demonstrate that it shares structural and functional properties with eukaryotic spectrins.

## Graphical abstract

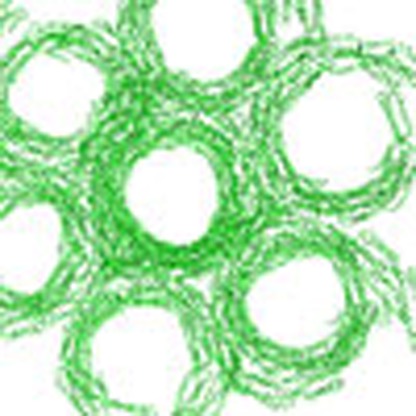

Bacterial cell division is directed by cytoskeletal proteins. The bacterial homologue of eukaryotic tubulin, FtsZ^1^, polymerises in the presence of GTP^2^ and is attached to the membrane at the mid-cell position to form the Z-ring^3^,^4^. The hydrolysis of GTP to GDP results in the dissociation of FtsZ polymers into monomers that disengage from the Z-ring^5^. Hence, the Z-ring is highly dynamic and constantly exchanges FtsZ molecules with the pool of its freely diffusible monomers^6^. The Z-ring is stabilized and anchored to the cytoplasmic membrane by interacting with FtsA, a bacterial homologue of actin, which is itself capable of polymerizing at the membrane^7^. FtsZ and FtsA recruit other cell division proteins, including cell wall synthesis enzymes and many proteins of uncharacterized function, to the mid-cell to form the mature divisome^8^. This large macromolecular assembly synthesizes the cell wall that will form the new poles of the cell after division^9^.

The polymerization of FtsZ into the Z-ring is a necessary and tightly regulated step in divisome assembly. The short C-terminal tail of FtsZ is essential for self-assembly and for interactions with its regulators^3^. Z-ring assembly is regulated by multiple, widely conserved cell division proteins, including FtsA, ZapA, the protease ClpX, SepF and EzrA. FtsA both recruits FtsZ filaments to the cell membrane and counteracts the clustering of FtsZ filaments on it^10^. ZapA and SepF promote the bundling of FtsZ filaments into higher-order structures *in vitro*^11^,^12^,^13^. SepF additionally stabilizes the Z-ring at mid-cell *in vivo*^14^ by binding to both FtsZ and the membrane^13^.

By contrast, the integral membrane protein EzrA was identified as an inhibitor of FtsZ polymerization and its absence results in the inappropriate formation of multiple FtsZ rings, not only at mid-cell but also at the cell poles^15^,^16^. However, EzrA is also one of the first proteins to be recruited to the mid-cell by FtsZ^8^, suggesting it has an additional regulatory or scaffolding role in the formation of the divisome. As an inhibitor of FtsZ polymerization, EzrA is assumed to be required to maintain the dynamics of the Z-ring as it constricts during cell division^16^. Other evidence supports a positive role for EzrA in the assembly of the divisome. In *Bacillus subtilis,* EzrA recruits the major bi-functional, glycosyltransferase-transpeptidase peptidoglycan synthase, penicillin-binding protein 1 (PBP1, *aka* PonA)^17^ to the septum. Bacteria require the synthesis of new peptidoglycan to grow, and peptidoglycan must be deposited as cells divide to ensure a continuum of cell wall around both halves of the dividing cell. Consequently, PBPs that synthesize new cell wall material must localize to areas of cell growth or division. Deletion of *ezrA* changes the cellular localization patterns of both FtsZ and PBP1, causing marked growth defects and alterations in cell morphology, division and elongation^17^,^18^. Generally, *ezrA* knockouts display a delay in cell division, resulting in cells about twice as long as wild type in *B. subtilis*^13^,^15^,^19^ and larger, spherical cells in *Staphylococcus aureus*^18^. The *ezrA* knockout has been described as lethal to *S. aureus*^20^, *Streptococcus pneumoniae*^21^,^22^ and *Listeria monocytogenes*^23^, and thus constitutes a novel target for antibacterial agents. In *B. subtilis*, the concurrent knockout of *ezrA* with other divisome components including *zapA*^11^, *sepF*^14^,^17^ or *gpsB*^17^ causes severe growth defects. Therefore, EzrA plays key roles both as a regulator of cell division and in the maintenance of cell size and shape.

EzrA comprises a single, N-terminal *trans*-membrane (TM) spanning helix followed by an ~60-kDa cytoplasmic domain^15^, which sequence analysis suggests forms an α-helical coiled-coil. To understand further the role of EzrA during cell division, we report the crystal structure of the intact, cytoplasmic domain of *B. subtilis* (*Bs*) EzrA at 3.95 Å resolution. Its three-dimensional structure comprises five, ~100 amino-acid long repeats, each of which forms an anti-parallel triple helical bundle; these are arranged linearly to form a complete semi-circle with a diameter of 120 Å. The individual repeats and their linear head-to-tail arrangement are reminiscent of the spectrin family of eukaryotic cytoskeletal proteins that associate with actin filaments and membrane-bound proteins. Two copies of the spectrin repeats are also observed in a 2.6-Å resolution structure of a 20-kDa cytoplasmic fragment of EzrA from *S. aureus* (*Sa*), which has enabled direct molecular comparisons to be made between eukaryotic spectrin repeats and those found in EzrA. FtsZ-binding sites on the *Bs*EzrA arch are mapped and a binding site that inhibits FtsZ oligomerization is identified. We propose a role for EzrA that is analogous to that of spectrins in eukaryotes, in linking the dynamics of the bacterial cytoskeleton to the membrane-anchored components of the divisome, the proper localization of which is essential for the bacterial cell cycle.

## Results

### EzrA has a semi-circular structure

The crystal structure of *B. subtilis* EzrA lacking the N-terminal 21 residue TM helix (that is, *Bs*EzrA_22–562_) was solved by selenomethionine single wavelength anomalous dispersion (SAD) to a resolution of 3.95 Å. Despite extensive crystallization screening, the reliable diffraction limit could not be extended. However, through the use of highly redundant data, the experimentally phased electron density maps of *Bs*EzrA_22–562_ (Supplementary Fig. 1a) enabled the building of 96% of the 540 amino acids in the structure. The only regions that could not be built were six residues at the N-terminus and a loop between Ser177 and Asn190, which the sequence predicted to be disordered. Although only a few side chain features are visible at this resolution, the final refined model, which includes sidechains, has geometric and crystallographic refinement parameters that compare very favourably with other structures of similar resolution (Table 1). The final model correctly aligns methionine residues with the experimentally determined positions of the selenium atoms from the SAD analysis (Supplementary Fig. 1b).

**Figure 1:**
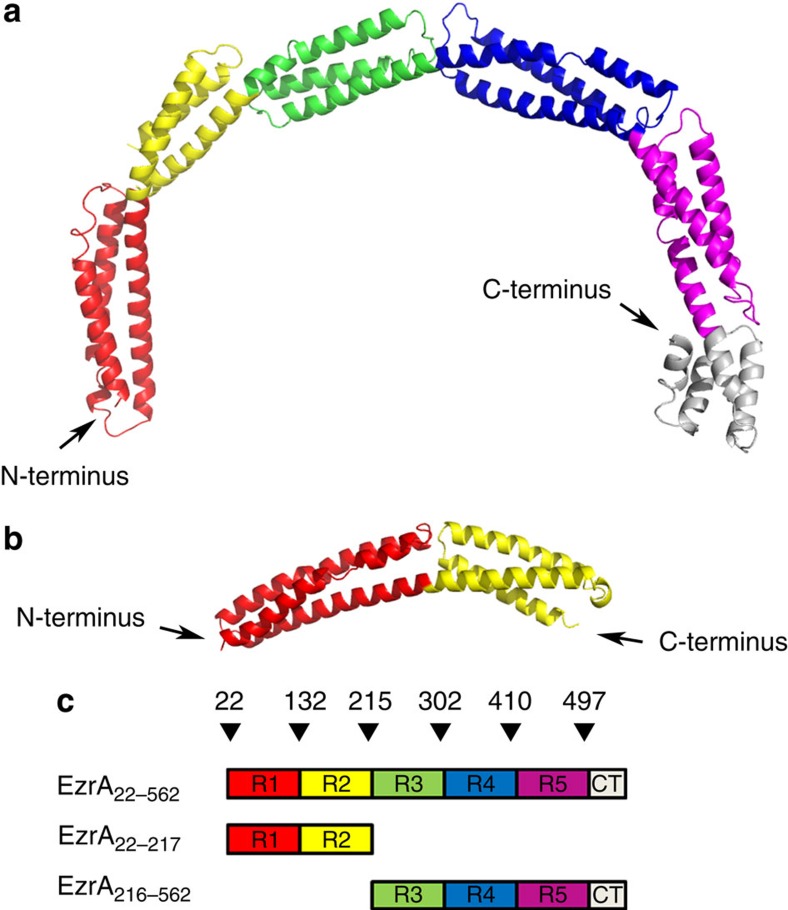
Structure of the cytoplasmic domain of EzrA. (**a**) Ribbon diagram of the *Bs*EzrA_22–562_ monomer coloured by triple helical bundle repeats from N- to C-terminus, red, yellow, green, blue and magenta. The C-terminal, four-helix bundle is coloured silver. This and all other structure representations were made in PyMOL (http:// www.pymol.org). (**b**) Ribbon diagram of the *Sa*EzrA_24–214_ monomer, coloured as in **a**. (**c**) Schematic illustration of the *Bs*EzrA fragments used in this study, with blocks (numbered R1–R5) representing the spectrin repeat units and the C-terminal four-helix bundle (labelled CT) and coloured as in **a**. The N- and C-termini and the junctions between spectrin repeats are numbered above the schematic.

Overall, the *Bs*EzrA_22–562_ monomer is a curved rod, forming a complete semicircle with a diameter of 120 Å. Groups of three α-helices pack together along its length (Fig. 1a), except at the C-terminus where there is a four-helix bundle. The formation of a semicircle confers the potential of the C-terminal four-helix bundle to interact with the membrane at the same time that the N-terminus of EzrA is embedded in it through its N-terminal TM helix; this can be modelled trivially by manually extending the N-terminal α-helix of repeat 1 (Supplementary Fig. 1c). An anti-parallel dimer is observed in the *Bs*EzrA_22–562_ crystal lattice as a result of crystallographic symmetry (Supplementary Fig. 2a). The dimerization of *Bs*EzrA_22–562_ in the crystal buries some 3,300 Å^2^ of surface area (~9% of the total) in an extensive, molecule-long interface. The anti-parallel dimer could not be detected in solution by sedimentation velocity, which indicates that the monomer and an oligomer of unknown arrangement are in equilibrium (Supplementary Fig. 2b). However, bacterial two-hybrid analysis *in vivo* shows an interaction between the N- and C-terminal regions of *Bs*EzrA (Supplementary Fig. 2c), which is consistent with the anti-parallel dimer seen in the crystals and which is the only oligomeric arrangement in the crystal that would enable the N-terminal TM helices of both subunits to insert into the same face of a membrane *in vivo*.

To obtain higher resolution structural information on EzrA, several fragments from different EzrA homologues were subjected to crystallization. A 190 residue N-terminal fragment of *S. aureus* EzrA, *Sa*EzrA_24–214_ (that also lacks its N-terminal TM helix), yielded crystals that diffracted to 2.6 Å resolution. Its structure (Fig. 1b, Supplementary Fig. 1d) was solved by molecular replacement using model phases from the equivalent portion of *Bs*EzrA_22–562_, and was refined to a final crystallographic *R*_free_ of 0.25 (Table 1). The structures of *Sa*EzrA_24–214_ and *Bs*EzrA_22–562_, which share only 20% sequence identity, superpose with root mean square deviations (RMSDs) of 2.4 Å on 154 paired Cα atoms (Supplementary Fig. 3a). The *Bs*EzrA_22–562_ and the shorter *Sa*EzrA_24–214_ rods comprise multiple copies of the same ~100 amino acid, triple helical bundle; *B*sEzrA_22–562_ contains five such repeats and *Sa*EzrA_24–214_ contains just two. The individual repeating units can all be superimposed with RMSDs of between 1.5 and 2.3 Å on paired Cα atoms (Supplementary Fig. 3b).

### Structural homologues of EzrA occur only in eukaryotes

A search for structural homologues of *Bs*EzrA_22–562_ using PDBefold^24^, DALI^25^ and BLAST-3D^26^ using default matching criteria did not identify any structures that superimposed convincingly along the entire length of the structure; only short segments could be aligned onto structural homologues. Representative matches from a BLAST-3D search included the eukaryotic cytoskeletal proteins vinculin^27^ and α-actinin^28^, the nuclear importers karyopherin^29^ and importin^30^ and four-helical coiled coils such as the bacterial chemotaxis receptor, CheA^31^ (Supplementary Fig. 4a). The same searches were repeated with the structure of *Sa*EzrA_24–214_ as the query. PDBefold identified five matches, all of which resembled the hits from the BLAST-3D search with *Bs*EzrA_22–562_; one match was to vinculin and the other four were to members of the spectrin superfamily to which α-actinin belongs.

Although EzrA structures could be aligned partially onto other proteins that also have an up-down-up arrangement of helices, the match with the spectrin superfamily of structures stood out as most significant. Despite having an up-down-up helix configuration, vinculin, karyopherin, importin and CheA all lack discrete triple-helical bundles; in all of these protein families, each helix packs intimately against three, rather than two, other helices. In addition, although DALI searches matched both EzrA structures with other rod-shaped triple helical bundles, such as BAR domains^32^ (Supplementary Fig. 4a), in these proteins there is only a single, triple helical bundle that is longer (>150 amino acids) than the repeat unit in EzrA. The linear head-to-tail arrangement of interconnected ~100 amino-acid triple helical bundles appears to be unique to spectrins and to EzrA. Finally, no matter which search tool was used, the C-terminal four-helix bundle of EzrA superimposed only weakly on matching structures in the PDB, but it is notable that it has a superficial resemblance to the helical bundle fold of the actin-binding calponin homology domains^33^, which can be found at the termini of spectrin-type proteins.

### EzrA is the founding member of the bacterial spectrin family

**Figure 2:**
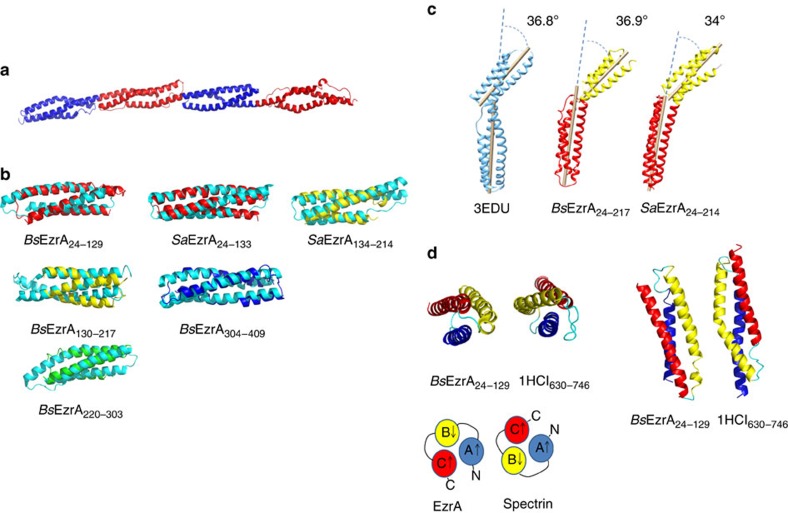
Comparison of EzrA with spectrin family proteins. (**a**) Structure of the spectrin protein α-actinin (PDB 1HCI), with alternating triple helical bundle spectrin repeats coloured red and blue. (**b**) Superimposition of spectrin units from *Bs*EzrA_22–562_ or *Sa*EzrA_24–214_ (coloured red, yellow, green and blue, as in Fig. 1a) onto representative spectrin units coloured cyan from erythroid spectrin (top; PDB 3KBT), α-actinin (middle; PDB 1HCI) and brain spectrin (bottom; PDB 1U5P). (**c**) The angle between spectrin repeats 1 and 2 in *Bs*EzrA_22–562_ is 37° and in *Sa*EzrA_24–214_ it is 34°; the angle is also 37° between erythrocytic β-spectrin repeats 14 and 15 (PDB 3EDU). (**d**) Top—Orthogonal views of the relative positioning and different connectivity of the three α-helices in EzrA spectrin repeat 1 (residues 24–129) and in the fourth repeat (residues 630–745) of the rod domain of α-actinin (PDB 1HCI). The N-terminal helix in the bundle (**a**) is coloured blue, the middle helix (**b**) yellow and the C-terminal helix (**c**) red. Bottom—Schematic illustration of the different connectivity in the EzrA and spectrin three-helix bundles, viewed along the helix axes. An up arrow (helix **a**, **c**) represents an ‘up’ helix viewed along the helix axis in an N–C direction (N-terminus of helix axis above plane of page). A down arrow represents a ‘down’ helix viewed in the opposite direction.

To our knowledge, the structural similarity of EzrA or any other bacterial protein to spectrins had not been predicted. From the logic below, we conclude that EzrA is the founding member of a new family of bacterial spectrin-like proteins: (i) the eukaryotic structural homologues of EzrA share the same extended rod-like character, comprising repeating units of ~100 amino acids arranged as triple helical anti-parallel bundles in a head-to-tail linear array (Fig. 2a, Supplementary Fig. 4b). Eukaryotic spectrins contain 4–20 of these units, called spectrin repeats, whereas EzrA contains 5 of them. (ii) Four of the five *Bs*EzrA_22–562_ spectrin repeats and the two in *Sa*EzrA_24–214_ can be superimposed on representative eukaryotic spectrin repeats with RMSDs of between 1.9 and 2.6 Å on matched Cα atoms (Fig. 2b); this is a high degree of similarity that is comparable to RMSDs among the EzrA spectrin repeats. Moreover, each superimposition accounts for greater than 70% of the EzrA spectrin repeat with paired amino acids coming from all three of the helices. (iii) The structural similarity of EzrA to the spectrin superfamily extends to the junctions and interfaces among adjacent repeats. As with all other crystallized spectrins, the repeat junctions in EzrA are formed from long continuous α-helices that form the third helix of one repeat and the first helix of the next. As also observed in several spectrins^34^,^35^,^36^, the *Sa*EzrA_24–214_ structure reveals intimate interactions at the interface between repeats involving the linker helix and loop residues. For example, the linker residues Tyr135 and Ile131 interact with loop residues Thr62, Leu56 and Tyr193 (Supplementary Fig. 5a,b). (iv) Although there are multiple examples of spectrin structures containing dual repeats, arranged like EzrA in a head-to-tail linear arrangement (Figs 1a and 2a, Supplementary Fig. 4b), the semi-circular curvature (Figs 1a and 2a) of *Bs*EzrA_22–562_ is unique. The principal axes of adjacent repeats in *Bs*EzrA_22–562_ intersect with angles of 37, 38, 33 and 53°, resulting in the pronounced overall bend. The angle between the two repeats in *Sa*EzrA_24–214_ is 34°, suggesting that full-length EzrA from *S. aureus* will also have a pronounced bend. The magnitude of these angles in EzrA is similar to that between erythrocytic β-spectrin repeats 14 and 15 (ref. 35), which is 37° (Fig. 2c). Furthermore, a study of the six-repeat plakin domain of desmoplakin in solution by small-angle X-ray scattering was consistent with the plakin adopting an overall L-shape^37^, but *Bs*EzrA_22–562_ is the first structure with a sufficient bend between enough repeats to generate a full semi-circle. The absence of longer chains of spectrin repeats from the PDB may simply reflect a crystallization bias towards the shorter, relatively rigid, dual spectrin repeats that predominate in the database.

Nevertheless, there are facets of the EzrA structure that differentiate it from spectrins. For instance, there is no sequence homology between EzrA and any spectrin, although short segments of both proteins share a heptad repeat periodicity of hydrophobic amino acids (Supplementary Fig. 6). Although the orientations of the three α-helices in the EzrA repeats are similar to those of spectrin repeats (Fig. 2a,b), the relative positions and therefore the connectivities of the helices are different (Fig. 2d). For example, the first ‘up’ helix in the EzrA repeat, helix A (blue, Fig. 2d), is superimposed on the third ‘up’ helix in the spectrin repeat from α-actinin, helix C (red, Fig. 2d). The second ‘down’ helix in the EzrA repeat is superimposed well on the α-actinin equivalent (helix B, coloured yellow in Fig. 2d), whereas the third ‘up’ helix in the EzrA repeat, helix C, is superimposed on the first ‘up’ helix in the α-actinin spectrin repeat, helix A. In other words, the left-handedness of the connectivity between helices in eukaryotic spectrin repeats (helices A–C are arranged in a counter-clockwise manner when viewed end-on) is reversed to be right-handed in EzrA (helices A–C are arranged clockwise).

### Direct interaction of EzrA with FtsZ

**Figure 3:**
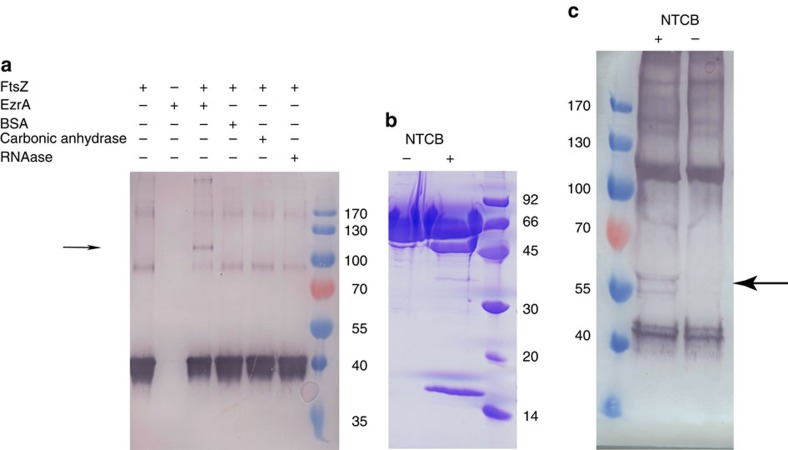
Qualitative binding of EzrA spectrin repeat 1 to FtsZ. (**a**) EDC crosslinking of *Bs*FtsZ to *Bs*EzrA_22–562_. *Bs*FtsZ with a 15-residue recombinant N-terminal T7 epitope tag was incubated with *Bs*EzrA_22–562_ or various control proteins with the crosslinking agent EDC. The crosslinking reactions were analysed by SDS–PAGE and by western blotting to detect the T7 epitope tag. Only the reaction containing *Bs*EzrA_22–562_ and *Bs*FtsZ yields a unique product (highlighted with an arrow) with a mobility consistent with a 1:1 EzrA_22–562_:FtsZ adduct of mass ~100 kDa. The units of the masses listed at the side of this and the other two panels in this figure are in kDa. (**b**) Cysteine-specific fragmentation of the *Bs*EzrA–*Bs*FtsZ adduct between spectrin repeats 1 and 2. The *Bs*EzrA_22–562_ mutant K135C/C222A has only one cysteine, at the interface between spectrin repeats 1 and 2. NTCB cleavage of isolated, purified K135C/C222A generates fragments of the expected size (16 kDa for residues 22–134 plus the N-terminal hexahistidine expression tag; 49 kDa for residues 135–562) on SDS–PAGE. (**c**) Cleavage of the *Bs*EzrA_22–562_K135C/C22A–*Bs*FtsZ crosslinked adduct with NTCB. The cleavage product was analysed by SDS–PAGE and by western blotting to detect the T7 epitope tag on FtsZ. After NTCB cleavage, a band (marked with an arrow) appears close to the 55-kDa marker, which is consistent with the 16-kDa N-terminal *Bs*EzrA fragment (residues 22–134) crosslinked to the 40-kDa *Bs*FtsZ.

To map which regions of *Bs*EzrA interact with *B. subtilis* FtsZ (*Bs*FtsZ), a qualitative crosslinking approach using 1-ethyl-3-(3-dimethylaminopropyl)carbodiimide (EDC), which conjugates amines directly to carboxyl groups, was combined with quantitative surface plasmon resonance (SPR) measurements. EDC-mediated zero-length crosslinks were found between *Bs*FtsZ and the *Bs*EzrA_22–562_ fragment, but not to unrelated control proteins (Fig. 3a). To determine more precisely the crosslinking site within EzrA, the *Bs*EzrA_22–562_–*Bs*FtsZ adduct was cleaved site-specifically into defined fragments using the cysteine-specific cleavage reagent 2-nitro-5-thiocyanatobenzoic acid (NTCB). For this, a *Bs*EzrA_22–562_ variant (K135C/C222A) with a single cysteine between spectrin repeats 1 and 2 was prepared (Fig. 3b). Cleavage of the *Bs*EzrA_22–562_(K135C/C222A)–*Bs*FtsZ adduct with NTCB yielded a fragment consistent with the attachment of *Bs*FtsZ to the N-terminal region of *Bs*EzrA (residues 22–134) comprising the first spectrin repeat (Fig. 3c).

**Figure 4:**
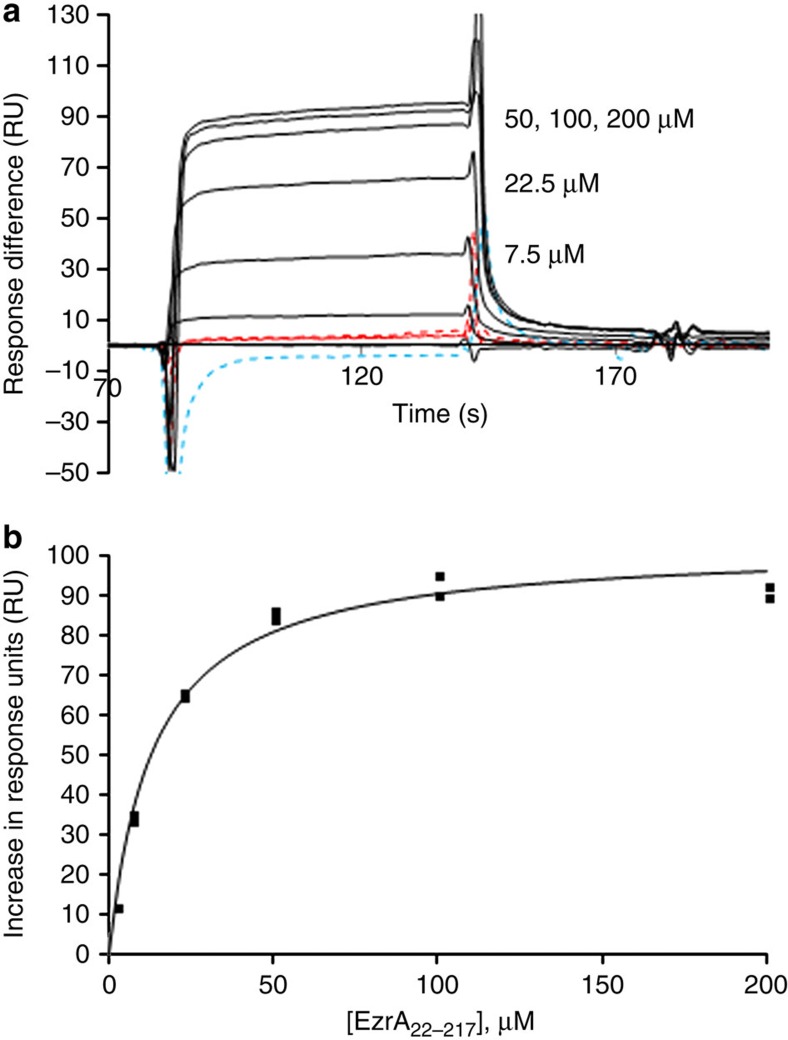
Quantification of binding of EzrA spectrin repeats 1–2 to FtsZ. (**a**) Binding of *Bs*EzrA_22–217_ to immobilized *Bs*FtsZ was monitored by SPR. Solid black sensorgrams represent the titration of increasing amounts of *Bs*EzrA_22–217_ over a chip on which *Bs*FtsZ was immobilized by amine coupling. The dashed cyan sensorgram represents an injection of 50 μM bovine serum albumin (BSA) over the same surface under the same conditions; the dashed red sensorgrams represent injections of 25, 50 and 100 μM of the *Bs*EzrA_131–217_ fragment. (**b**) The response units of *Bs*EzrA_22–217_ binding the *Bs*FtsZ surface as a function of injected protein were fit with a curve corresponding to a 1:1 binding interaction, yielding a *K*_d_ of 13.7±1.5 μM. The actual binding process may be more complex than a simple 1:1 interaction because of the self-association of the *Bs*EzrA_22–217_ fragment, apparent from size exclusion chromatography-multi angle laser light scattering analysis (SEC-MALLS) (see Supplementary Fig. 7b).

Guided by limited proteolysis studies and the crystal structure, two truncated *Bs*EzrA fragments were produced. *Bs*EzrA_22–217_ encompassed repeats one and two and *Bs*EzrA_216–562_ included the other three and the C-terminal domain (Fig. 1c). *Bs*EzrA_22–217_ bound to an SPR chip surface coated with immobilized *Bs*FtsZ with fast kinetics, enabling equilibrium binding levels to be measured (Fig. 4a). Fitting the binding isotherm to a 1:1 binding model yielded a dissociation constant (*K*_d_) of ~13 μM for the *Bs*FtsZ:*Bs*EzrA_22–217_ complex (Fig. 4b). The *Bs*EzrA_216–562_ fragment also bound to the *Bs*FtsZ-coated chip, but with considerably slower kinetics (Supplementary Fig. 7a), and fitting the sensorgrams to a 1:1 binding model yielded a *K*_d_ of 2 μM. The specificity of the observed interactions is supported by the lack of any interaction of either *Bs*EzrA_131–217_ or bovine serum albumin with the FtsZ chip surface under equivalent buffer conditions (dashed cyan sensorgrams, Fig. 4a and Supplementary Fig. 7c).

The binding of both *Bs*EzrA_22–217_ and *Bs*EzrA_216–562_ to *Bs*FtsZ implies that there are either two distinct FtsZ-binding sites on an EzrA monomer or a single site that extends across the interface between repeats 2 and 3. However, to be consistent with the crosslinking of *Bs*FtsZ to repeat 1, a single binding site at the interface between repeats 2 and 3 would need to extend also to spectrin repeat 1, implying an intimate association of FtsZ with repeat 2. However, a fragment encompassing only repeat 2, *Bs*EzrA_131–217,_ showed no detectable interaction with immobilized *Bs*FtsZ (red dashed traces, Fig. 4a) in SPR, despite it having appreciable secondary and tertiary structure content (Supplementary Fig. 8a,b). Therefore, it seems most likely that there are at least two distinct FtsZ interaction sites on an EzrA monomer, one of which is in spectrin repeat 1.

### Effect of EzrA on FtsZ oligomerization

**Figure 5:**
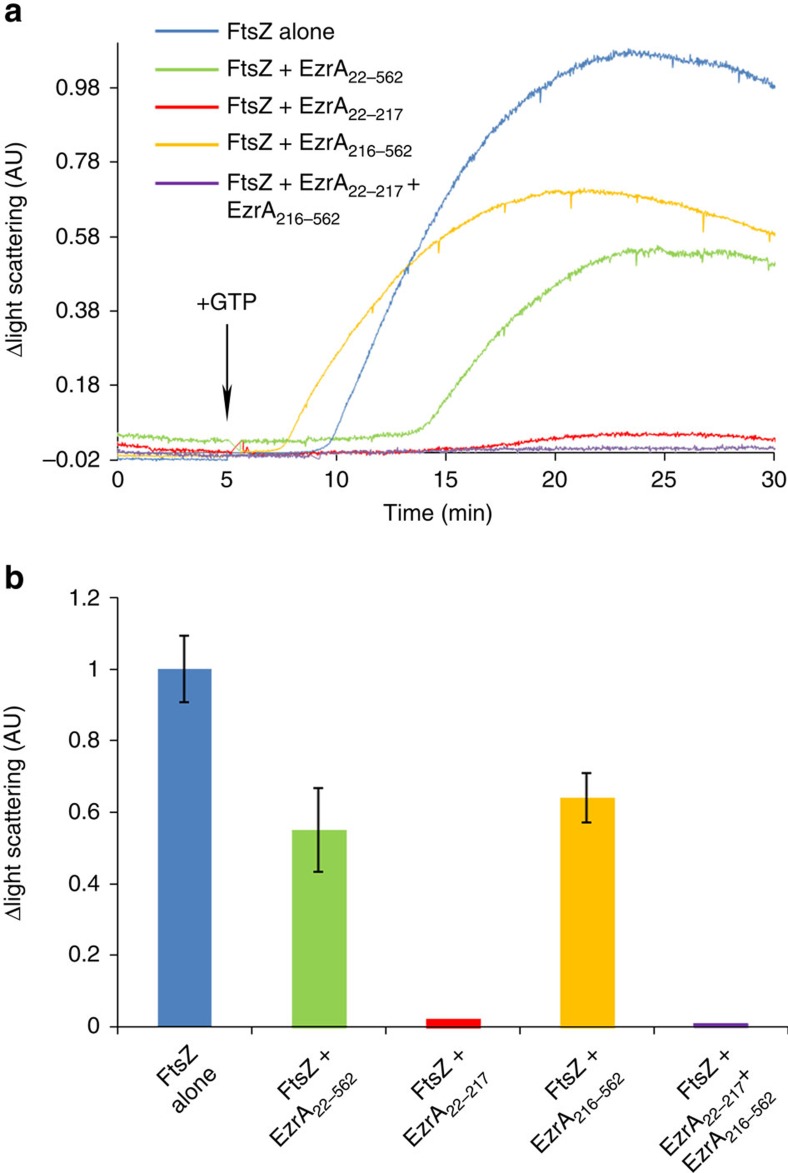
Inhibition of FtsZ oligomerization by EzrA fragments. (**a**) The GTP-induced oligomerization of FtsZ was monitored by light scattering. All samples contained 12.5 μM *Bs*FtsZ and 25 μM of the indicated *Bs*EzrA fragment. The arrow corresponds to the time when GTP was added to a final concentration of 1 mM. (**b**) The average GTP-induced increase in light scattering in the presence of the *Bs*EzrA fragments. The results shown are the mean±s.d. from three independent measurements. The *Bs*EzrA_22–217_ fragment, encompassing spectrin repeats 1–2, reduces FtsZ polymerization to undetectable levels.

The *Bs*EzrA–*Bs*FtsZ interaction was analysed to map regions of EzrA responsible for the inhibition of FtsZ oligomerization. GTP-induced increase in light scattering provides a measure of the polymerization of FtsZ monomers into filaments and the bundling of filaments into higher order structures; we refer to these processes collectively as ‘oligomerization’. Previous studies have shown that a twofold excess of *Bs*EzrA over *Bs*FtsZ reduced FtsZ oligomerization between two- and tenfold^16^,^38^. The pH was increased from 6.5, which was used in previous studies with this assay, to 7.3, to reduce the aggregation of *Bs*EzrA_22–562_. Under these conditions, a twofold excess of *Bs*EzrA_22–562_ inhibited *Bs*FtsZ oligomerization about twofold (Fig. 5a,b), consistent with previous studies^16^,^38^. Remarkably, identical concentrations of *Bs*EzrA_22–217_ in this assay inhibited *Bs*FtsZ oligomerization almost completely (Fig. 5a,b), whereas a twofold excess of *Bs*EzrA_216–562_ reduced *Bs*FtsZ oligomerization by about one-third. An equimolar mixture of *Bs*EzrA_22–217_ and *Bs*EzrA_216–562_ also completely inhibited *Bs*FtsZ assembly (Fig. 5a,b). The stronger inhibition of *Bs*FtsZ assembly by the smaller *Bs*EzrA_22–217_ fragment in comparison to *Bs*EzrA_22–562_ may reflect the masking of FtsZ-interacting sites by the oligomerization of *Bs*EzrA_22–562_; size exclusion chromatography of *Bs*EzrA_22–562_ revealed that it formed an oligomer in the low salt buffer conditions used in the light scattering assay (Supplementary Fig. 8c). The relative levels of inhibition of *Bs*FtsZ assembly by different *Bs*EzrA fragments must be interpreted cautiously, but the marked inhibition by *Bs*EzrA_22–217_ points once more to a functionally important interaction of the N-terminal region of EzrA with FtsZ.

To ascertain whether the marked inhibition of *Bs*FtsZ oligomerization by *Bs*EzrA_22–217_ reflects direct inhibition of FtsZ polymerization into filaments, or the blocking of filament bundling into higher order structures, its effect on the GTPase activity of *Bs*FtsZ was examined. Head-to-tail polymerization of FtsZ into filaments is dependent upon GTP binding^39^, and dissociation of FtsZ polymers into monomers is dependent upon GTP hydrolysis^5^. Therefore, either the inhibition of GTP binding or the stimulation of the GTPase activity could result in the inhibition of FtsZ head-to-tail polymerization by EzrA. The presence of *Bs*EzrA_22–217_ did not markedly affect the binding of the fluorescent BODIPY-GTP-γ-S to *Bs*FtsZ (Supplementary Fig. 9a), a result that is consistent with the very slight reduction (1.6-fold) in GTP affinity for *Bs*FtsZ in the presence of *Bs*EzrA measured previously^40^. The GTPase activity of *Bs*FtsZ was measured in the presence and absence of *Bs*EzrA_22–217_, to determine whether EzrA affected GTP hydrolysis and thus FtsZ oligomerization. Both *Bs*EzrA_22–562_ and *Bs*EzrA_22–217_ had marginal effects on GTP turnover in a coupled GTPase assay (Supplementary Fig. 9b) and in a malachite green assay that monitored release of free phosphate (Supplementary Fig. 9c). It would thus seem that EzrA has little impact on the binding of GTP by FtsZ or on its hydrolysis.

Finally, we examined the impact of *Bs*EzrA_22–217_ on the oligomerization of *Bs*FtsZ_1–366_, which lacks the C-terminal 17 amino acids required for bundling of FtsZ filaments^41^,^42^. Consistent with previous observations^41^, the change in light scattering on adding GTP to *Bs*FtsZ_1–366_ was reduced markedly, by ~15-fold relative to wild-type *Bs*FtsZ (Supplementary Fig. 10a). As has been observed previously with *Bs*EzrA_22–562_ (ref. 43), EzrA_22–217_ had no impact on the residual GTP-induced oligomerization activity of *Bs*FtsZ_1–366_ (Supplementary Fig. 10). These results suggest that EzrA does not affect the head-to-tail polymerization of FtsZ into filaments but instead largely acts by preventing the bundling of FtsZ filaments into higher-order assemblies.

## Discussion

Spectrins are ubiquitous proteins involved in the organization of the cytoskeleton and membrane processes in animals. A typical spectrin has an elongated molecular shape due to multiple, connected repeats of three anti-parallel α-helices, which are slightly curved and coil around each other. Individual spectrin repeats are approximately 50 Å long and 20 Å wide and are similar in length (99–122 amino acids). Their primary amino-acid sequences differ significantly, but the bundling of the helices is stabilized by regularly spaced hydrophobic side chains. EzrA shares all of these features with the spectrin superfamily of proteins, and the structural similarity of the EzrA repeats with those of other spectrin-like proteins is striking (Fig. 2). Although many proteins are known to comprise bundles of antiparallel α-helices, EzrA is, to the best of our knowledge, the only bacterial protein that is formed from serial spectrin-like repeats.

Structure-based sequence alignments do not show any meaningful sequence homology either among the five EzrA repeats, or between EzrA and spectrins. Such a lack of sequence conservation is not without precedence within the spectrin superfamily^44^. The spectrin repeat is a degenerate sequence established predominantly on the heptad repeat pattern of hydrophobic amino acids in the primary sequence, a characteristic of left-handed helical supercoiled proteins^45^. The degeneracy is exemplified by the observation that there are only four invariant residues in an alignment of the four spectrin repeats in human α-actinin 2, one invariant residue in an alignment of the ten spectrin repeats in the human Duchenne muscular dystrophy protein and none in the seventeen spectrin repeats of human erythrocyte β-spectrin. Therefore, even the tryptophan in the first helix in the repeat, originally described as critical for thermodynamic stability of the spectrin repeat^46^,^47^, is not strictly maintained. The first and third repeats in EzrA each contain a highly conserved proline residue in helix A; Pro45 of *Sa*EzrA in repeat 1 (Supplementary Fig. 11a) is conserved in eight out of nine phylogenetically diverse paralogues, whereas Pro228 (Pro227 in *Bs*EzrA) in repeat 3 (Supplementary Fig. 11b) is conserved in all of them. Although sequence analyses of spectrins have identified that a proline is found frequently in helix B^48^, neither helix A nor C has been reported to be interrupted by proline residues.

There is significant variation in the relative orientation of adjacent repeat units in spectrin structures^35^,^49^. However, EzrA is the first spectrin-like repeat-containing structure in which there is a pronounced enough bend between a sufficient number of repeats to form a complete semicircle. Molecular dynamics studies suggest the flexibility of the junction regions in spectrins is constrained appreciably, with the force constants for changing the angle between adjacent repeats determined^36^ to be between 20 and 60 kcal mol^−1^ rad^−2^. A considerable input of energy is thus likely to be necessary to convert the semicircular EzrA into a straight rod. The solvent contents of the EzrA crystals are unusually high (*Bs*EzrA_22–562_ 80%; *Sa*EzrA_24–214_ 74%); >80% of all entries in the PDB have a solvent content <60% and only 1.1% have a solvent content >75%. The high solvent content of these crystals does not concur with the release of sufficient energy during crystallization to drive thermodynamically unfavourable events. Therefore, the semicircular shape is highly unlikely to represent an artefact induced by crystal packing. The extended curved structure is a property EzrA shares with SepF, an FtsZ bundling protein required for normal septum formation. *B. subtilis* SepF assembles into polymeric arcs^13^ and rings^12^ with a diameter (55 nm) larger than that of the EzrA semicircle (12 nm). FtsZ filaments are proposed to bind on the outside of the SepF curve, whereas the inner face interacts with the surface of the invaginating septum membrane^13^. An analogous intimate association of the much smaller inner face of the EzrA semicircle with the membrane *in vivo* would require extreme curvature of the lipid bilayer, which has not been reported to date.

A question raised by the *Bs*EzrA_22–562_ structure is the orientation of full-length EzrA relative to the lipid bilayer. A tilted orientation of the EzrA arch with respect to the membrane could result from the curvature of the membrane, the bending of the TM helix or flexibility in the junction between the TM helix and the cytoplasmic domain. The conserved proline in helix 1 of repeat 1 (Pro45 in *S*. *aureus* EzrA) may confer some additional flexibility in the junction between the body of the cytoplasmic domain and the membrane. However, an intimate association of the EzrA cytoplasmic domain with the surface of the membrane, as seen in the curve-shaped helical BAR domain proteins^32^, is not supported by electrostatic potential calculations on either of the EzrA structures presented here. A tilted orientation of the arch would position the C-terminal four-helix bundle away from the surface of the membrane. This domain contains a conserved motif between residues 505 and 511, termed the ‘QNR patch’ (Supplementary Fig. 1c), that is important for mid-cell localization^38^. The QNR patch has been proposed recently to bind directly to FtsZ, based upon the inhibition of FtsZ oligomerization by different EzrA fragments in a light scattering assay *in vitro*^50^.

In the simplest model for the orientation of EzrA, the TM helix would form a straight extension of the first few turns of helix A of the first spectrin repeat; secondary structure predictions suggest a continuous uninterrupted helix extends into the first helix in the cytoplasmic domain. This orientation would result in the cytoplasmic domain of EzrA forming an arch with the C-terminal helical bundle positioned close to the membrane surface (Supplementary Fig. 1c). Although the sequence register in this region of the structure must be regarded cautiously, because of the paucity of methionine residues in this region of the sequence, this model would position key solvent-exposed residues in the QNR patch (R510, R512 and F511) to interact with the membrane, and preclude their direct interaction with Z-ring FtsZ polymers (Supplementary Fig. 1c). This suggests rather that the QNR patch controls EzrA localization through direct interaction with the membrane or specific membrane-associated protein(s).

**Figure 6:**
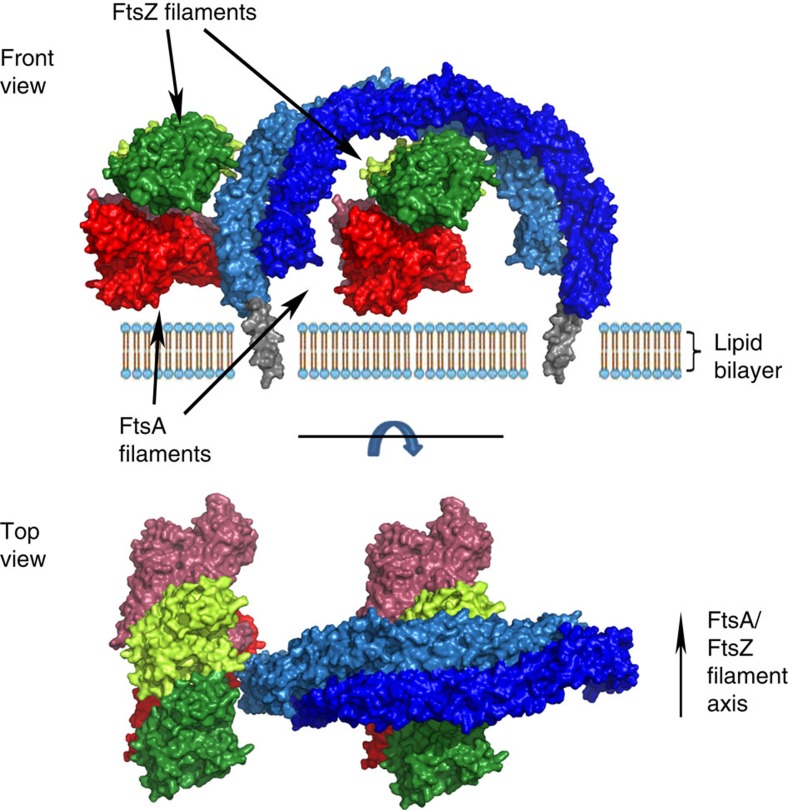
Model of the interaction of EzrA with the cytoskeleton at the membrane surface. Model of FtsZ and FtsA protofilaments situated both alongside and beneath the inner face of the arch-shaped EzrA molecule (here represented as the crystallographic anti-parallel dimer, component subunits coloured light and dark blue). The modelled TM helix of EzrA is coloured grey. The coordinates of *T. maritima* FtsA bound to a C-terminal fragment of FtsZ (PDB 4A2A) and the *M. jannaschii* FtsZ dimer (PDB 1W59) and *B. subtilis FtsZ* monomer (PDB 2VAM) were used to model FtsA (light and dark red) and FtsZ (light and dark green) protofilaments, respectively. FtsA has been oriented to place the membrane-anchored C-terminus close to the lipid bilayer and its binding site for FtsZ near the FtsZ filament. The FtsZ filament has been positioned to place its C-terminal FtsA-binding region (disordered in FtsZ crystal structures) close to the FtsA filament. An FtsZ filament trapped beneath the EzrA arch cannot bundle together with filaments outside the arch. Although the crystallographic dimer is represented, the same effect on bundling could be made by EzrA monomers.

An arch over the membrane could allow EzrA to regulate FtsZ oligomerization by trapping FtsZ filaments in the gap between the membrane and the inside of the EzrA curve. Molecular modelling with the *Methanococcus janaschii* FtsZ dimer^51^ and the *Thermotoga maritima* FtsA:FtsZ complex^7^ reveals there is sufficient space beneath the arch, in both monomeric and dimeric forms of EzrA, to accommodate an FtsZ filament or even to simultaneously accommodate FtsA-mediated membrane-anchored FtsZ and FtsA protofilaments (Fig. 6). Trapping FtsZ protofilaments inside the arch is intuitively an effective means to disrupt lateral interactions between protofilaments and could additionally anchor contractile protofilaments to the membrane. An individual filament inside the arch cannot engage in lateral interactions with filaments outside of it (Fig. 6), providing an additional level of Z-ring regulation. The ability of the arch to accommodate FtsZ protofilaments could be modulated depending on the cellular location of EzrA by the curvature of the cell membrane and/or by competing interactions of EzrA with other divisome components such as GpsB. In addition, or alternatively, FtsA and FtsZ may interact with the outside face of the EzrA arch only, leaving the inner surface for mediating alternative protein–protein interactions.

A bacterial origin for the eukaryotic cytoskeleton has been suggested previously, in part, because of the similarity in structure and function of FtsZ to tubulin and FtsA to actin, although the primary amino-acid sequences are poorly related^1^,^52^,^53^ and their structures differ in some respects. For example, two C-terminal α-helices (residues 381–437), which cover the surface of tubulin and are intimately associated with the globular body of the protein, are not visible in many FtsZ structures from different organisms^53^,^54^,^55^,^56^,^57^ or are replaced by β-strands^51^. In addition, the relative positioning of the four subdomains differs in FtsA and actin^58^, and these two proteins are further distinguished by the unique presence in FtsA of subdomain 1C^58^. Our study extends the similarities between the eukaryotic and prokaryotic cytoskeletons to proteins that contain spectrin-like repeats. An established function of spectrins in eukaryotes is to connect actin filaments to other membrane-associated proteins and, at least in the case of plectin, to also regulate actin dynamics^59^. An analogous role can thus be envisaged for EzrA during cell division, co-ordinating FtsA/FtsZ dynamics inside the cell to peptidoglycan biosynthesis on the outside, an activity that is essential for cytokinesis and which is effected by the recruitment of PBP1 to the site of division by its interaction with EzrA^17^.

## Methods

Full details of the Methods are supplied in the Supplementary Information.

### Expression plasmid construction

The genes encoding fragments of *Bs* and *Sa*EzrA and T7 epitope-tagged FtsZ were cloned into pET-based vectors. The plasmids pBS58 and pCXZ for overexpressing FtsZ were a gift from David Adams (Newcastle University).

### Expression and purification of EzrA and EzrA fragments

Recombinant EzrA proteins were produced using *E. coli* strain BL21(DE3) carrying the appropriate plasmid. Expression was induced in Luria Bertani (LB) medium with isopropyl β-D-1-thiogalactopyranoside or by auto-induction. For the isopropyl β-D-1-thiogalactopyranoside -induced expression of selenomethionine-labelled proteins, the methionine-auxotrophic host strain B834 (DE3) was grown in a defined, minimal medium based on M9 supplemented with appropriate ions, vitamins and 19 amino acids; methionine was replaced by selenomethionine. For the *Bs*EzrA proteins, the cells were harvested by centrifugation, before the cell pellet was resuspended in lysis buffer. Cells were lysed by sonication and clarified by centrifugation. The supernatant was filtered and then purified by Ni-nitrilotriacetic acid (Ni-NTA) affinity chromatography with elution effected by imidazole. The hexahistidine tag was removed by overnight incubation with thrombin, before further purification by size exclusion chromatography. The purified proteins were snap-frozen in liquid nitrogen for storage. *Sa*EzrA_24–214_ was produced without a tag, and was purified by ammonium sulphate fractionation and anion exchange chromatographic steps.

### Expression and purification of *Bs*FtsZ

Untagged *Bs*FtsZ was co-expressed with *E. coli* FtsQ, FtsA and FtsW using plasmids pCXZ and pBS58, as described previously^60^. FtsZ was purified by ion exchange chromatography and serial ammonium sulphate precipitation at 20, 30 and 40% (w/v). The proteins precipitated with 40% ammonium sulphate were resuspended and purified further by size exclusion chromatography, before snap-freezing in liquid nitrogen for storage. T7-tagged *Bs*FtsZ was purified by Ni-NTA affinity chromatography following the same procedures as used for His-tagged *Bs*EzrA fragments, and the His-tag was removed by thrombin.

### Crystallization and structure determination

*Bs*EzrA_22–562_ was crystallized using a mother liquor of 50 mM MES/NaOH, pH 6.7, 1 M tri-sodium citrate, 0.1 M ammonium sulphate, 0.2 M non-detergent sulphobetaine-201. The structure of EzrA was solved by single wavelength anomalous dispersion from a single selenomethionine-labelled crystal. The heavy atoms’ substructure and initial phasing were determined in SHELX^61^. SHELXE^61^ and PHASER^62^ were used for subsequent density modification. The atomic model was built by manually docking α-helices into the electron density in COOT^63^. The amino-acid sequence was assigned by matching the positions of selenium atoms to methionine positions in the protein sequence. SLOOP^64^ was used to find loops that best fit the electron density map. The geometry of the loop–helix junctions was corrected using RAPPER^65^ and PHENIX. REFINE^66^ was used for refinement of the model.

*Sa*EzrA_24–214_ was crystallized in a buffer of 34% 2-methyl-2, 4-pentanediol, 0.1 M Tris-HCl, pH 7.5, and its structure was solved by molecular replacement using equivalent atoms from the *Bs*EzrA_22–562_ structure as a search model in PHASER^62^ and refined to convergence using COOT^63^ and REFMAC5 (ref. 67). Summaries of the data collection and model refinement statistics are provided in Table 1, and Molprobity^68^ was used for structure validation reports.

### EDC crosslinking

EzrA fragments were mixed with T7-tagged FtsZ and cross-linked with EDC before analysis by SDS–polyacrylamide gel electrophoresis (SDS–PAGE) and western blotting. To map the crosslink site on EzrA, crosslinked proteins were purified from non-crosslinked proteins by Ni-NTA before NTCB cleavage, SDS–PAGE and immunoblotting with an anti-T7 antibody.

### Light scattering

Light scattering measurements used a Varian Cary Eclipse fluorimeter at 30 °C, with all samples in a buffer of 50 mM Tris–HCl, pH 7.3, 20 mM KCl, 3 mM magnesium acetate. Samples were pre-incubated at 30 °C, centrifuged in a microfuge and the supernatant transferred to a quartz microcuvette. GTP was added to a final concentration of 1 mM and light scattering recorded for at least 30 min. The GTP-induced change in light scattering in the presence of EzrA and FtsZ was normalized relative to the average GTP-induced scattering change for FtsZ alone.

### Surface plasmon resonance

All SPR experiments used a Biacore X100 instrument with the chip surface at 25 °C. Proteins were immobilized on Biacore CM5 chip surfaces by standard protocols. The binding data were all fit using the Biacore X-100 evaluation software; for affinity fits, the response on the surface 4 s before the end of the injection were fit as a function of the concentration of the injected EzrA fragment. Each SPR titration shown was performed independently at least twice.

### GTPase assay

The GTPase activity of FtsZ was measured under the same buffer conditions as used for the light scattering assays. In an NADH oxidation-coupled continuous regenerative assay^69^, 150 μl samples were incubated at 30 °C in a microplate reader after the addition of GTP to 1 mM. NADH consumption was measured, which is coupled to GDP production, from the decrease in absorption at 340 nm. For malachite green assays, the release of inorganic phosphate was measured 20 min after adding GTP to 2 mM. A standard curve was generated using phosphate ions in the concentration range 50–450 μM.

### GTP binding

Fluorescence was measured at 22 °C in a Varian Cary Eclipse fluorimeter. 150 μl of 250 nM BODIPY GTP-γ-S in 50 mM HEPES, pH 7, 100 mM KCl, 5 mM MgCl_2_ was preincubated for >5 min before adding 4–10 μl of a stock solution of either 8 μM FtsZ or 8 μM FtsZ with 16 μM *Bs*EzrA_22–217_ in the same buffer.

## Additional information

**Accessions codes.** The atomic co-ordinates and the associated structure factors have been deposited in the Protein Data Bank (http:// www.pdbe.org) with accession code 4UXV for *Bs*Ezr_A22–562_ and 4UY3 for *Sa*EzrA_24–214_.

**How to cite this article:** Cleverley, R. M. *et al.* Structure and function of a spectrin-like regulator of bacterial cytokinesis. *Nat. Commun.* 5:5421 doi: 10.1038/ncomms6421 (2014).

## Supplementary Material

Supplementary Information Supplementary Figures 1-11, Supplementary Tables 1-2, Supplementary Methods and Supplementary References

## Figures and Tables

**Table 1:** Crystallographic statistics.

	***Bs*** **EzrA**_**22–562**_	***Sa*** **EzrA**_**24–214**_
*Data collection*		
Space group	*R*32	*P*4_3_2_1_2
Cell dimensions		
*a*, *b*, *c* (Å),	222.1, 222.1, 183.5	96.1, 96.1, 97.1
*α*, *β*, *γ* (°)	90, 90, 120	90, 90, 90
Resolution (Å)	29.2–3.95 (4.16–3.95)*	48.56–2.6 (2.72–2.6)*
*R*_sym_	0.104 (0.351)	0.081 (0.972)
*R*_pim_	0.03 (0.266)	0.031 (0.368)
*I*/σ*I*	21.3 (3.6)	15.3 (2.8)
Completeness (%)	99.6 (99.2)	100 (100)
Redundancy	22.4 (4.0)	15.3 (2.8)
Anomalous completeness (%)	99.2 (95.7)	NA
Anomalous redundancy (%)	11.4 (2.5)	NA
Solvent content (%)	82.2	74.7
*Refinement*		
Resolution (Å)	29.2–3.95	48.56–2.6
No. of reflections	15,307	13,770
*R*_work_/*R*_free_	0.320/0.348	0.209/0.249
*No. of atoms*		
Protein	4,264	1,586
Ligand/ion	0	0
Water	0	0
*B*-factors		
Protein (Å^2^)	155.6	88.4
*RMSD*		
Bond lengths (Å)	0.003	0.0102
Bond angles (°)	0.785	1.34
Molprobity geometry score	2.6 (98th percentile)	1.6 (100th percentile)
Molprobity clash score	9.4 (97th percentile)	1.15 (100th percentile)

NA, not applicable; RMSD, root mean square deviation.

^*^Where values in parentheses refer to the highest resolution shell.
