# Supplementary material for: Structure and function of a spectrin-like regulator of bacterial cytokinesis
Source: Nat Commun. 2014 Nov 18;5:5421. doi: 10.1038/ncomms6421 (PMC4243239; doi:10.1038/ncomms6421)
Supplement: Supplementary Information — Supplementary Figures 1-11, Supplementary Tables 1-2, Supplementary Methods and Supplementary References [file ncomms6421-s1.pdf]

Supplementary Information for:

Structure and function of a spectrin-like regulator of bacterial cytokinesis

Robert M. Cleverley[1], Jeffrey R. Barrett[2], Arnaud Baslé[1], Nhat Khai Bui[1,†], Lorraine Hewitt[1], Alexandra Solovyova[3], Zhi-Qiang Xu[2], Richard A. Daniel[1], Nicholas E. Dixon[2], Elizabeth J. Harry[4], Aaron J. Oakley[2], Waldemar Vollmer[1] and Richard J. Lewis[1]

[1] Institute for Cell and Molecular Biosciences, Newcastle University, Newcastle upon Tyne, NE2 4HH, U.K

[2] Centre for Medical and Molecular Bioscience, University of Wollongong, NSW 2522, Australia

[3] NUPPA, Devonshire Building, Newcastle University, Newcastle upon Tyne, NE1 7RU, U.K

[4] The ithree institute, University of Technology, Sydney, NSW 2007, Australia

[†] Present address: Helmholtz Centre for Infection Research, Helmholtz Institute for Pharmaceutical Research, Saarland University, 66123 Saarbrücken, Germany

Correspondence: R.J.L: r.lewis@ncl.ac.uk

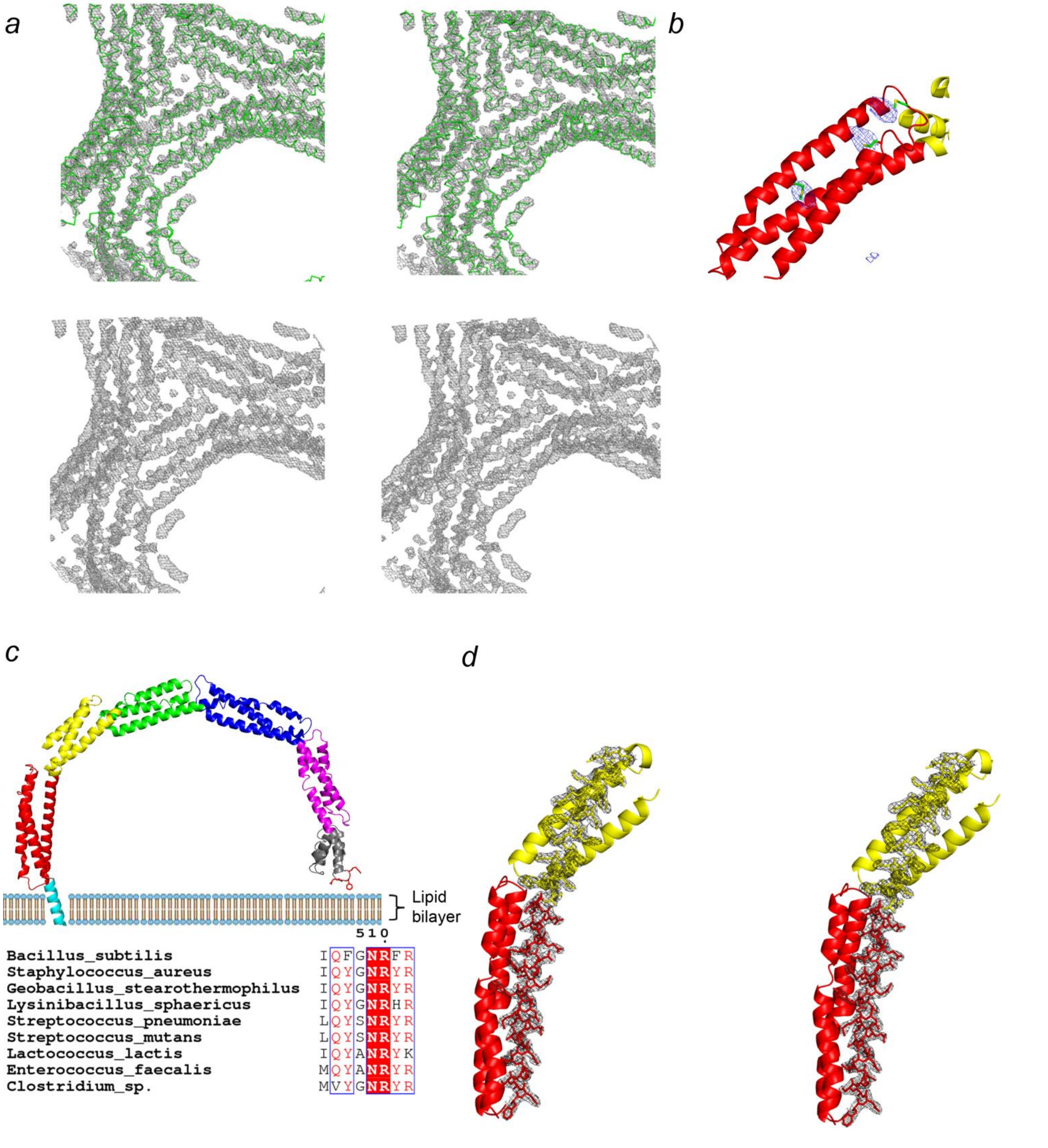

Supplementary Figure 1: Structures of *Bs*EzrA$_{22–562}$ and *Sa*EzrA$_{24–214}$.

(**a**) Experimentally phased SAD electron density map (black "chicken-wire" representation) contoured at a level of 2 σ. The top panels display in wall-eyed stereo the map and symmetry mates of the final refined structure of *Bs*EzrA$_{22–562}$, drawn as green C_α traces, whereas the bottom panel shows the electron density alone.

(**b)** Anomalous difference Fourier map calculated with model phases and contoured at a level of +5σ in the region of the first spectrin repeat of *Bs*EzrA$_{22–562}$ to show the correct assignment of the amino acid sequence. Side chains are represented as sticks for methionine residues.

(**c**) To model the position of the *trans*-membrane helix in full length EzrA, the N-terminus of *Bs*EzrA$_{22–562}$ (coloured red, yellow, green, blue, magenta and silver, as in **Fig. 1a**) has been extended with a standard straight α-helix, coloured cyan. The conserved residues R510, F511 and R512, which project from the C-terminal bundle towards the putative membrane surface, are shown in stick representation in red; the inset alignment shows R510 and R512 are highly conserved and an aromatic residue, predominantly tyrosine, is conserved at position 511.

(**d**) Electron density for the central longest helix (residues 101–161) in the *Sa*EzrA$_{24–214}$ structure, represented in wall-eyed stereo; the central helix is shown in stick representation and the remainder of the structure in cartoon form. The final, REFMAC-weighted $2F_{obs}–F_{calc}$ electron density map is contoured at 1.7σ.

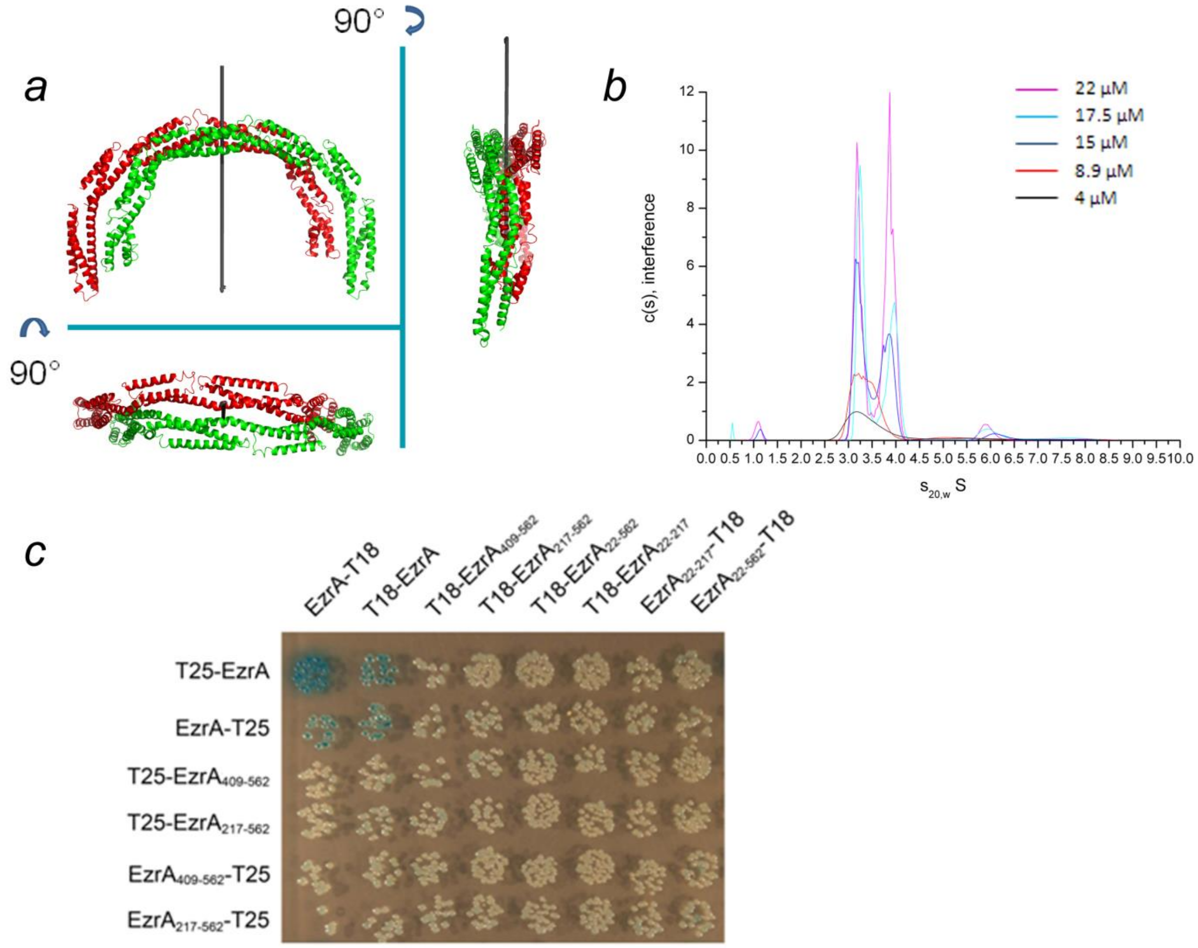

Supplementary Figure 2: EzrA self-assembly *in vitro* and *in vivo.*

(**a**) Ribbon representation of the *Bs*EzrA$_{22–562}$ dimer formed by crystallographic symmetry. Each protomer is coloured separately and the two-fold crystallographic axis of symmetry relating the two subunits of the dimer is shown in black.

(b) *Bs*EzrA$_{22–562}$ self-assembly *in vitro*. Sedimentation velocity analyses of *Bs*EzrA$_{22–562}$ at concentrations between 4 and 22 µM reveals two major species with sedimentation coefficients of 3.2 and 3.9 *S*, revealed by plotting the *c*(*s*) sedimentation velocity distribution for EzrA$_{22–562}$ at the five protein concentrations listed. The former species is the EzrA monomer, whereas the latter is an oligomer with the two species in equilibrium because the oligomer:monomer ratio increases as a function of protein concentration. The value of 3.2 *S* for the monomer is in good agreement with a theoretical value of 3.0 *S* calculated from the coordinates[1,2]. However, the value of 3.9 *S* for the oligomer deviates significantly from the value of 5.08 *S* calculated from the coordinates[1,2] of the anti-parallel homodimer; this oligomer must therefore have a more highly elongated overall shape than the anti-parallel dimer, with a less compact association of component subunits.

(**c**) EzrA self-assembly *in vivo*, monitored by bacterial two hybrid analysis. To probe whether anti-parallel dimerization of the full length, membrane associated *Bs*EzrA was favoured *in vivo*, the self-interaction of *Bs*EzrA was analysed by adenylate cyclase-based bacterial 2-hybrid analysis using constructs tagged at both the N- and C-termini. Constructs were prepared by fusing either the full length coding sequence of *ezrA* or specific truncations of *ezrA* to fragments of the *cya* adenylate cyclase coding sequence, T18 or T25. Fusions to the T25 fragment are on the left of the panel and T18 fusions are at the

top of each column. The position of the T18 or T25 fragment relative to the *ezrA* sequence is represented by this nomenclature: T25-EzrA$_{n\text{-}m}$ represents the fusion of the T25 coding sequence to N-terminus of an *ezrA* coding sequence spanning residues n and m; EzrA$_{n\text{-}m}$-T18 represents a fusion of the T18 coding sequence to the C-terminus of the relevant *ezrA* sequence. Notably, an interaction is only observed between full length EzrA fusion proteins and the strength of the EzrA-EzrA interaction is strongly dependent on the position of the reporter tags. A combination of N- and C-terminally tagged partners (*e.g.* T25-EzrA and EzrA-T18) gives a stronger interaction than the interaction between partners that have fusions at the same EzrA terminus (*i.e.* T25-EzrA and T18-EzrA, or EzrA-T25 and EzrA-T18). The implicit monotopic[3] topology of the T18-EzrA and T25-EzrA fusions, with both N and C-terminal ends of the EzrA TM helix in the cytoplasm, has been observed in other membrane proteins such as caveolin[4] and prostaglandin synthase[5], most likely in this instance because the N-terminal Cya fusion does not cross the membrane. The geometry of an elongated anti-parallel dimer, in which the N- and C-termini on interacting subunits are in close proximity (as observed in EzrA$_{22–562}$ crystals), is consistent with these two hybrid results.

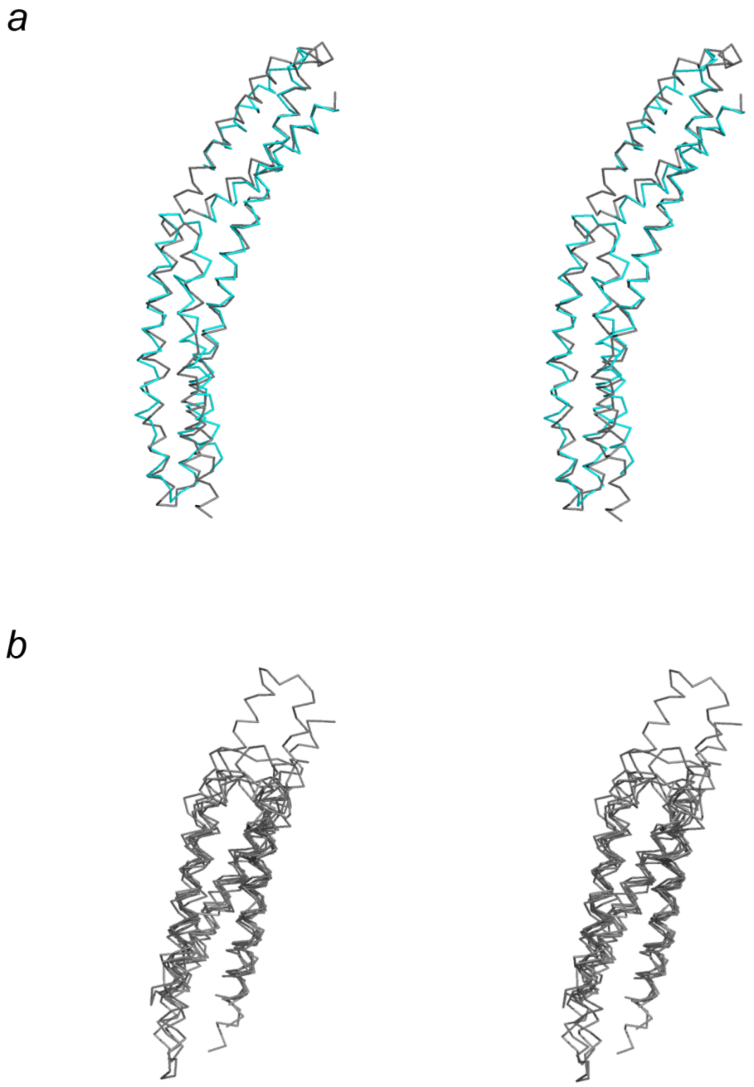

Supplementary Figure 3: Comparison of *Bs*EzrA$_{22\text{–}562}$ and *Sa*EzrA$_{24\text{–}214}$ structures.

(**a**) The views of a global superposition of equivalent residues from *Bs*EzrA$_{22\text{–}562}$ (cyan) and *Sa*EzrA$_{24\text{–}214}$ (grey) drawn as Cα-traces in wall-eyed stereo.

(**b**) Superposition of all 7 EzrA spectrin-like repeats, shown in wall-eyed stereo.

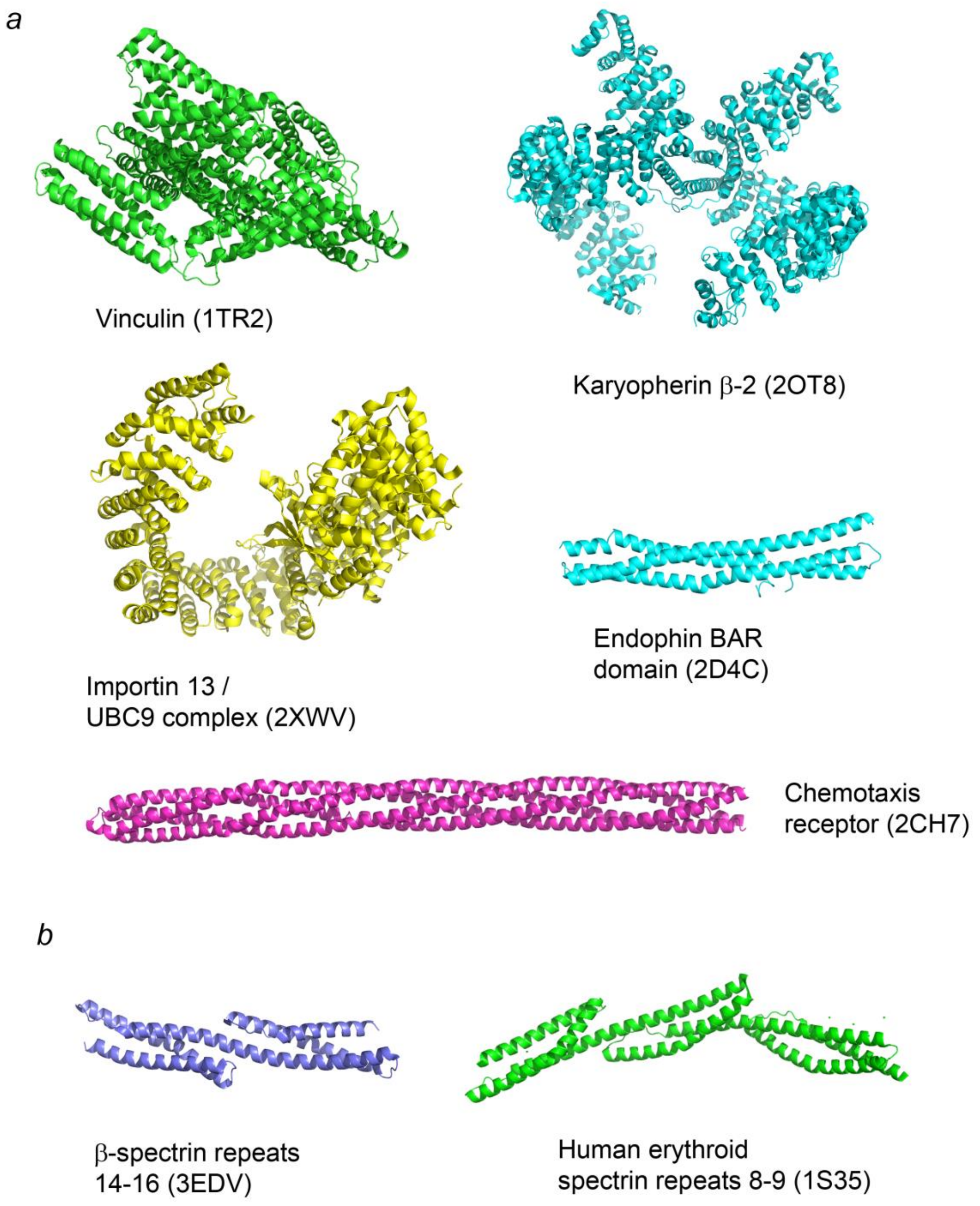

Supplementary Figure 4: Representative all α-helical protein structures

(**a**) Structures of vinculin (1TR2[6]), karyopherin (2OT8[7]), importin (2XWU[8]), the chemotaxis receptor (2CH7[9]) and endophilin BAR domain (2D4C[10]) are shown as cartoon models; these structures have a serendipitous and superficial likeness to EzrA on the sole basis of a shared propensity for adopting all α-helical folds with an antiparallel alignment of helices.

(**b**) Representative two- and three-spectrin repeat structures (1S35[11]; 3EDV[12]) are shown as cartoon models; the global similarity to EzrA can be seen by comparison to **Fig. 1a**.

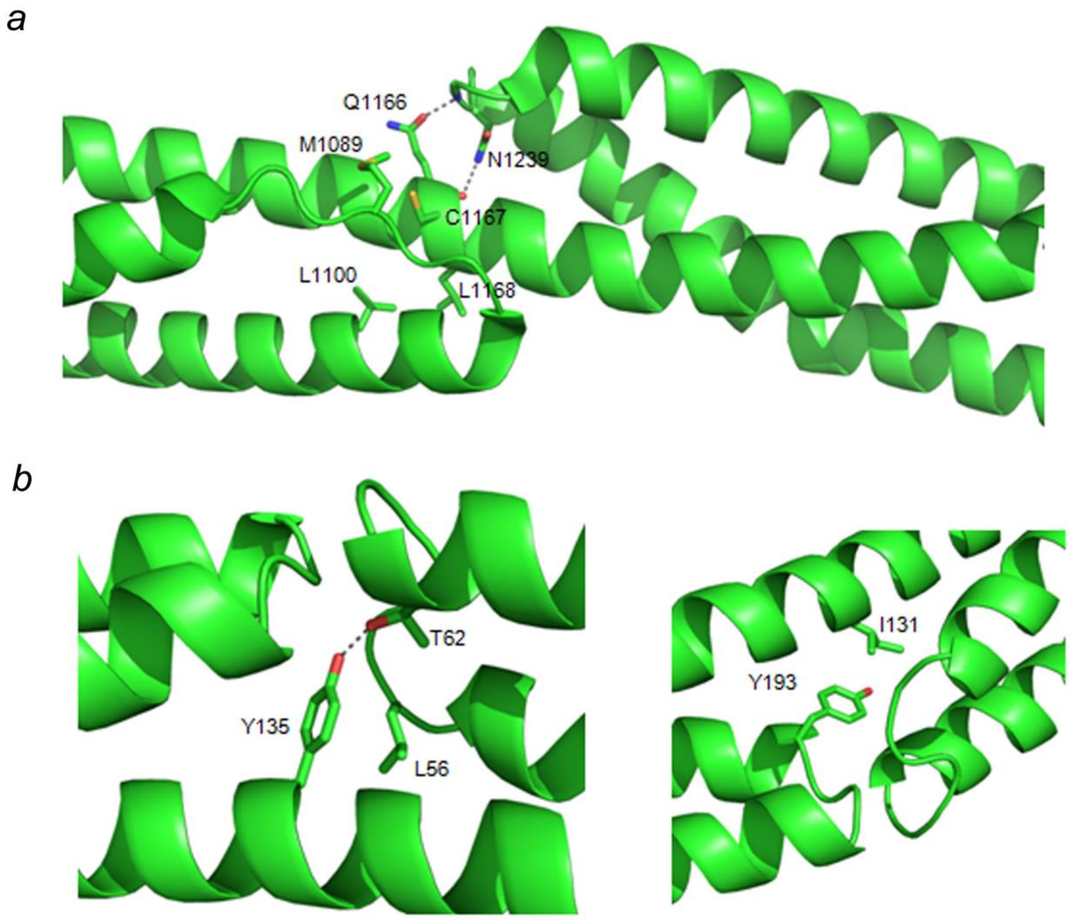

Supplementary Figure 5: Helix-loop interactions

(**a**) Helix-loop interactions in human erythroid spectrin (1S35[11]), a representative spectrin. The side chain amide oxygen of the linker helix Gln1166 hydrogen bonds to the main chain amide nitrogen of Leu1240 in the loop and the loop Asn1239 side chain amide nitrogen hydrogen bonds to the main chain carbonyl oxygen of Gln1166. Cys1167 and Leu1168 in the linker helix form van der Waals' contacts with the loop residue Met1089 and with Leu1100, which is positioned at the loop/helix interface. Dashed black lines represent hydrogen bonds.

(**b**) Interactions between residues at the interface between repeat units in *Sa*EzrA$_{24–214}$. Left: Tyr135, Thr62 and Leu56 are shown in stick representation; the dashed line represents the hydrogen bond between Tyr135 in the linker helix and Thr62 at the start of helix B in repeat 1. The aromatic ring of Tyr135 also packs against Leu56 in the loop between helices A and B in repeat 1. Right: Ile131 in the linker helix forms Van der Waals' contacts with the aromatic ring of Tyr193 in the loop between helices 2 and 3 in repeat 2.

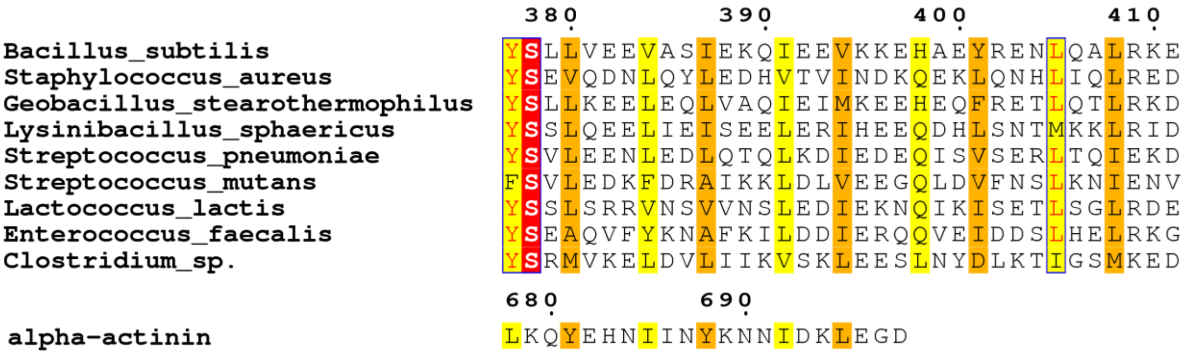

Supplementary Figure 6: Heptad sequence distribution of EzrA

The sequence segment represented here corresponds to the third helix in the fourth spectrin-like repeat unit of *B. subtilis* EzrA. The buried, typically hydrophobic residues *a* and *d* in each heptad repeat are boxed in yellow and orange, respectively. In the *Bs*EzrA$_{22–562}$ structure, the polar residue His398, occupying the *a* position, is buried, which is reconciled with it being in close proximity to the similarly buried Glu326. A representative heptad repeat pattern of hydrophobic amino acids in the α-helix of a representative spectrin repeat, from α-actinin, is also shown.

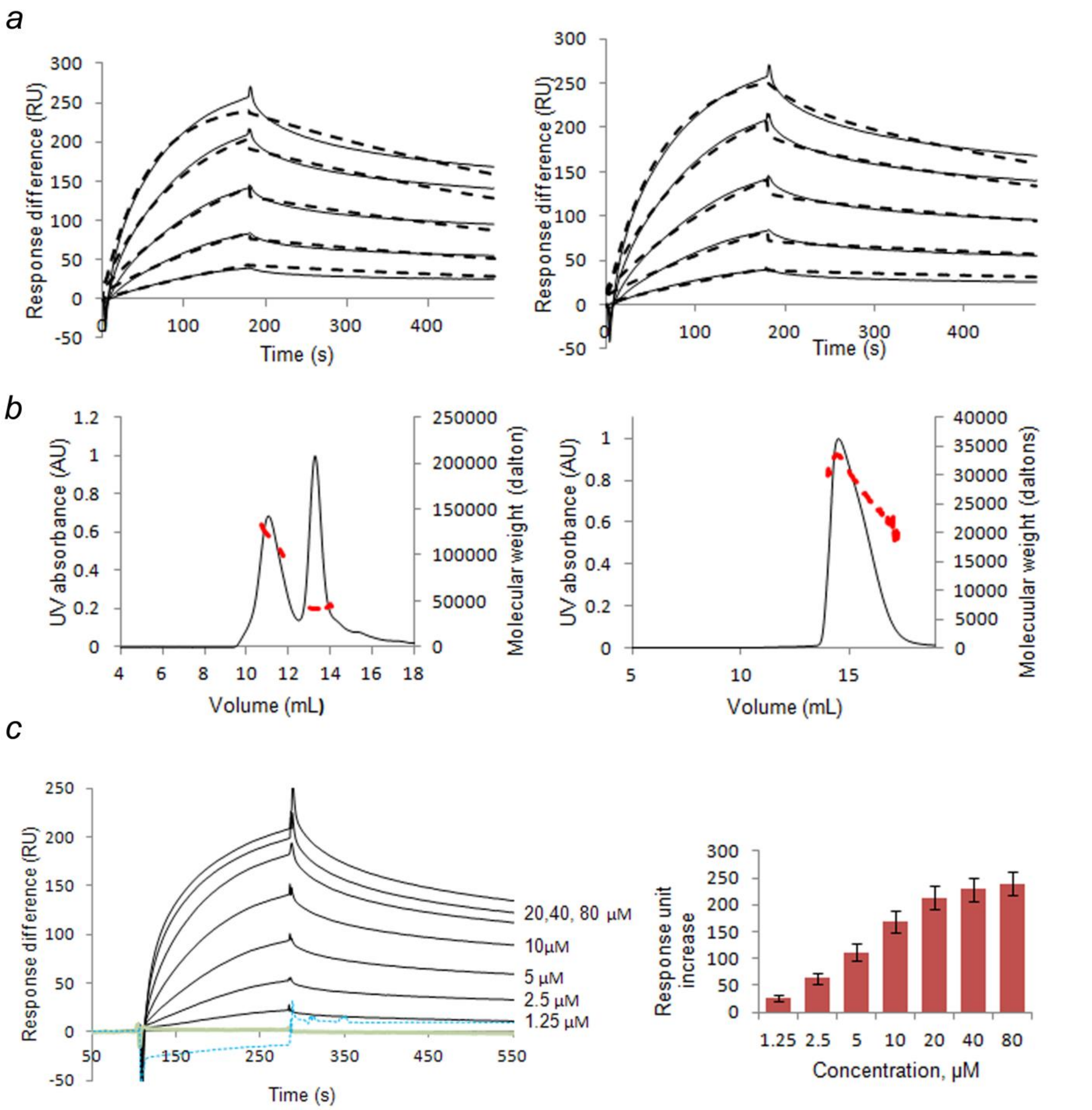

Supplementary Figure 7: *Bs*FtsZ binds to *Bs*EzrA$_{216\text{-}562}$ encompassing spectrin repeats 3-5 and the C-terminal four helix bundle.

(a) SPR sensorgrams for injection of various concentrations of *Bs*EzrA$_{216–562}$ (solid black lines) in 10 mM MES.NaOH, pH 6.5, 150 mM KCl over a chip surface on which *Bs*FtsZ was covalently immobilized. The sensorgrams have been fitted with kinetic binding constants according to a 1:1 binding model (left panel) or a bivalent analyte binding model (right panel) that considers a second, weaker binding site for *Bs*FtsZ on *B*sEzrA$_{216–562}$; the dashed traces correspond to the sensorgrams simulated with the fit parameters that are listed in **Supplementary Table 1**. The improved fit obtained with the bivalent analyte model may reflect the tendency of *Bs*EzrA$_{216–562}$ to oligomerize in solution as detected by SEC-MALLS (panel **b**). Although the 1:1 kinetic fit likely underestimates the complexity of the binding process, the magnitude of the affinity obtained from this fit (K_d <10 μM) is consistent with approximations of equilibrium binding levels from the raw sensorgrams in a titration (**panel c**).

(b) SEC-MALLS analysis of EzrA fragments *Bs*EzrA$_{216–562}$ (left) and *Bs*EzrA$_{22–217}$ (right). The solid line corresponds to UV absorbance (left axis) and the dashed red trace, overlaid on the principal peaks (between 10-12 and 13.5-14 mL for *Bs*EzrA$_{216–562,}$ 14-16 mL for *Bs*EzrA$_{22–217}$), represent the deconvoluted molecular weights of eluting species (right axis). For *Bs*EzrA$_{216–562}$ the peak between 13.5-14 mL is consistent with the monomer molecular weight of 39.9 kDa, while the broader peak between 10-12 mL represents an oligomer. *Bs*EzrA$_{22–217}$ has a monomer molecular weight of 23.8 kDa; the deconvoluted molecular weights across the broad 14–17 mL peak range from 34–23 kDa and most probably indicate a poorly resolved monomer and dimer.

(**c**) Pre-equilibrium binding levels for the *Bs*EzrA$_{216–562}$:*Bs*FtsZ interaction. SPR sensorgram (left panel) of successive *Bs*EzrA$_{216–562}$ injections (between 1.25 and 80 μM) over a *Bs*FtsZ-immobilised chip. The dashed cyan sensorgam represents the injection of 40 μM BSA over the same surface. The response units that bind the *Bs*FtsZ surface after each 180 s injection are plotted (right panel) as a function of injected protein concentration (average of three measurements ± standard error); the binding levels saturate at 20 μM injected protein concentration.

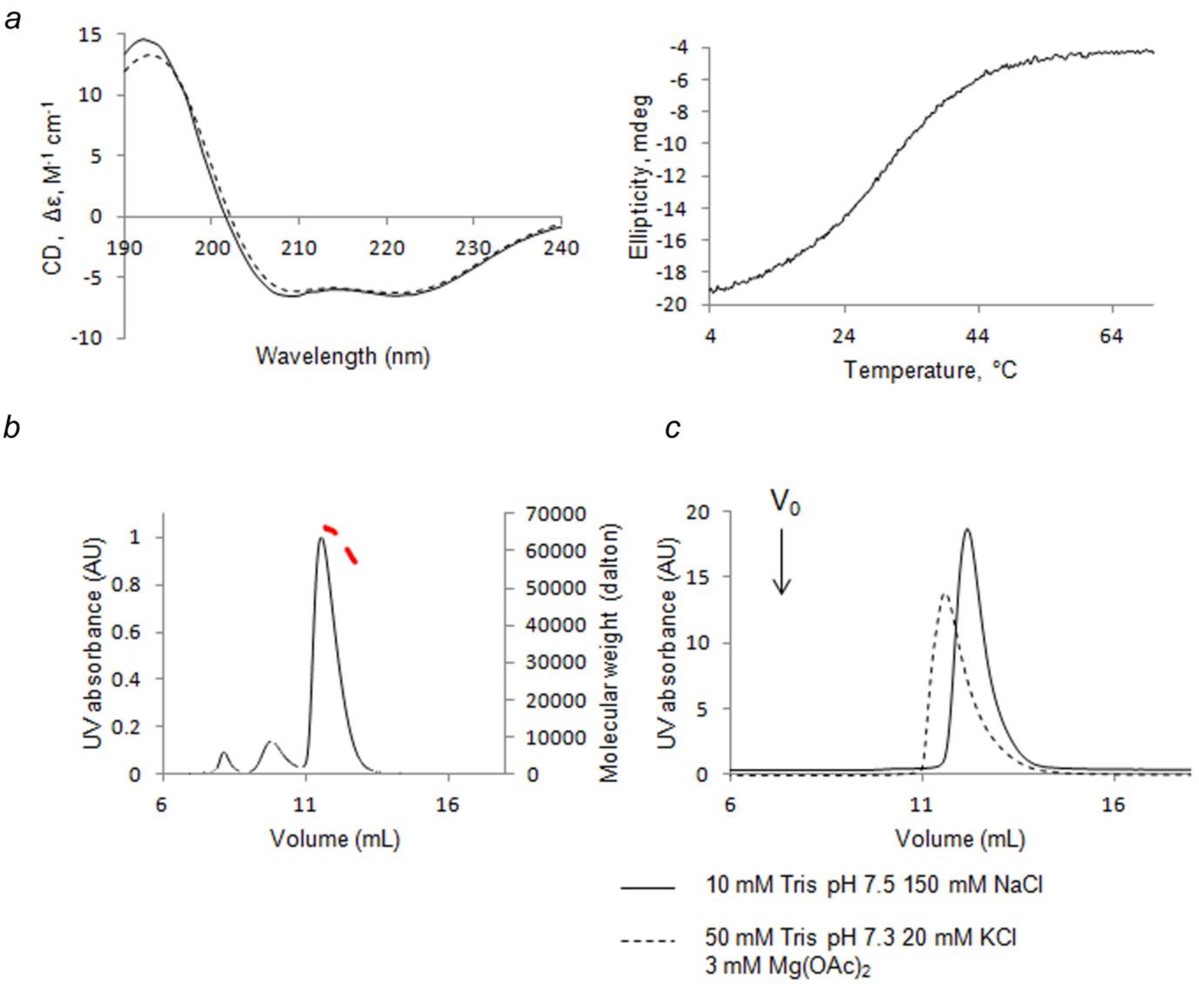

Supplementary Figure 8: Secondary and tertiary structure of *Bs*EzrA$_{131–217}$.

(**a**) The CD spectrum (left panel) of *Bs*EzrA$_{131–217}$ encompassing spectrin repeat 2 (solid line); a simulated spectrum is shown (dashed line, calculated with SELCON[13] in the DICHROWEB[14] server), which corresponds to an overall helical content of 67%. By comparison, the helical content for this region in the crystal structure, calculated with DSSP[15] is 65%. Thermal melt (right panel), monitoring ellipticity at 222 nm as a function of temperature. The melt has a sigmoidal character but with a broad transition and a low midpoint of 35°C, indicating that the tertiary structure of *Bs*EzrA$_{131–217}$ does not have great thermal stability.

(**b**) Oligomerization of *Bs*EzrA$_{22–562}$ in low salt buffers. SEC-MALLS analysis of *Bs*EzrA$_{222–562}$ in 10 mM Tris.HCl, pH 7.5, 150 mM NaCl revealed the predominant species, eluting at 12 mL, has a molecular weight consistent with the monomer (63 kDa). The solid black line, mapping to the left axis, represents UV absorbance and the dashed red line, mapping to the right axis, represents the deconvoluted molecular weights of eluting species.

(**c**) Comparative SEC analysis in 10 mM Tris.HCl, pH 7.5, 150 mM NaCl (solid line) and in the low salt, magnesium-containing buffer used in light scattering (50 mM Tris.HCl, pH 7.3, 20 mM KCl, 3 mM magnesium acetate, dashed line) shows a reduced elution volume in the low salt buffer, consistent with assembly into an oligomer. The arrow and V_o symbol denote the void volume of the Superdex200HR10/300 gel filtration column used here (7 mL).

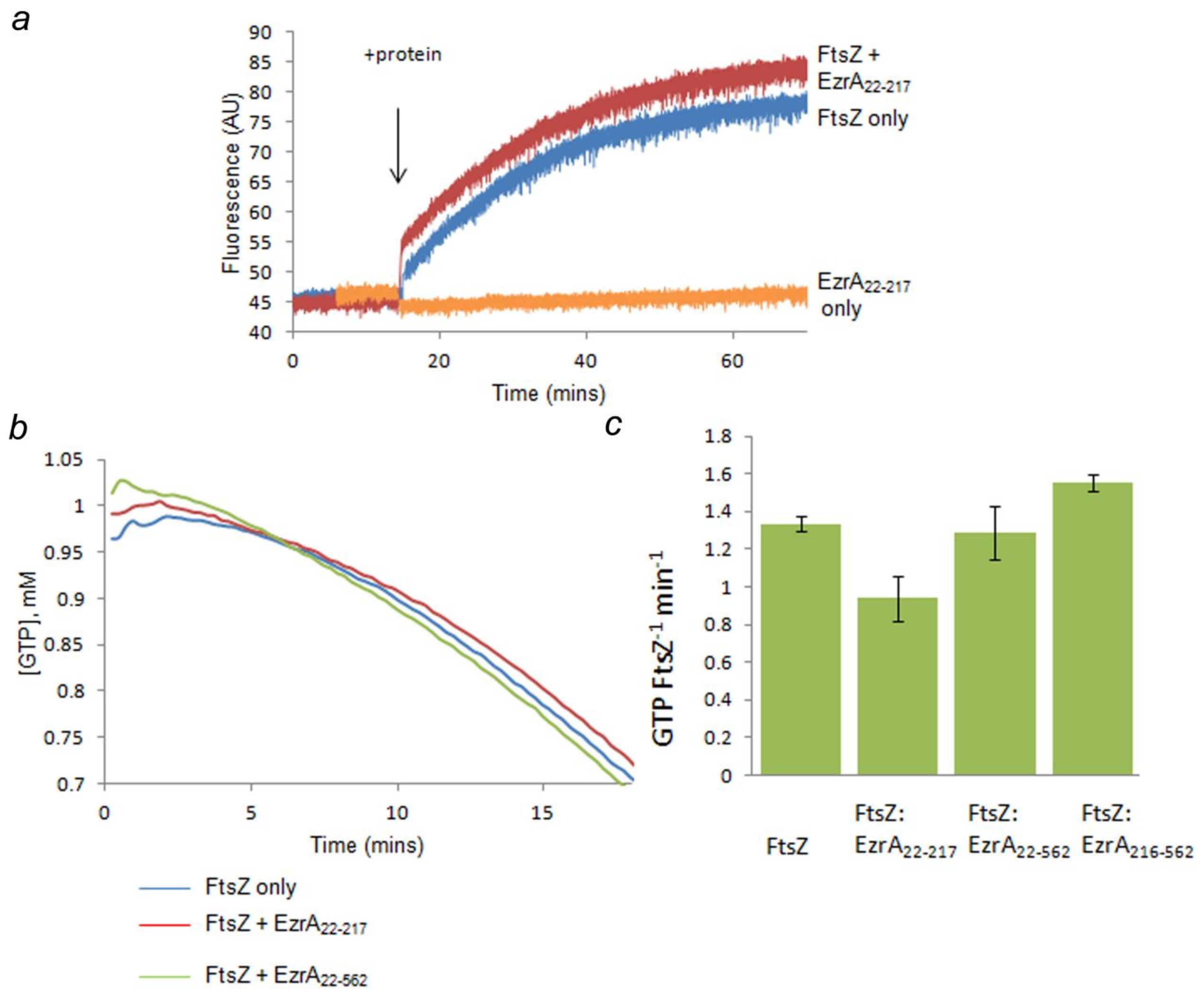

Supplementary Figure 9: *Bs*EzrA$_{22–217}$ has little effect on *Bs*FtsZ GTP binding and hydrolysis.

(**a**) Binding of a fluorescently-labelled GTP analogue, BODIPY-GTP-γ-S, to *Bs*FtsZ, monitored by an increase in fluorescence emission at 535 nm. The arrow corresponds to the time point of the addition of *Bs*FtsZ, or of a 1:2 mixture of *Bs*FtsZ and *Bs*EzrA$_{22–217}$ to the final concentrations indicated; BODIPY-GTP-γ-S is at 250 nM.

(**b**) GTPase activity of *Bs*FtsZ in the presence and absence of a two-fold excess of *Bs*EzrA$_{22–562}$ and *Bs*EzrA$_{22–217}$, monitored by an assay coupling GDP production to consumption of NADH by lactose dehydrogenase[16].

(**c**) GTPase activity of *Bs*FtsZ in the presence and absence of a two-fold excess of various EzrA fragments, monitored by a malachite green assay measuring the release of inorganic phosphate. The GTPase activities are based on the phosphate released after 20 min incubation with GTP; overall, the different EzrA fragments have only marginal effects on GTP turnover within this timeframe.

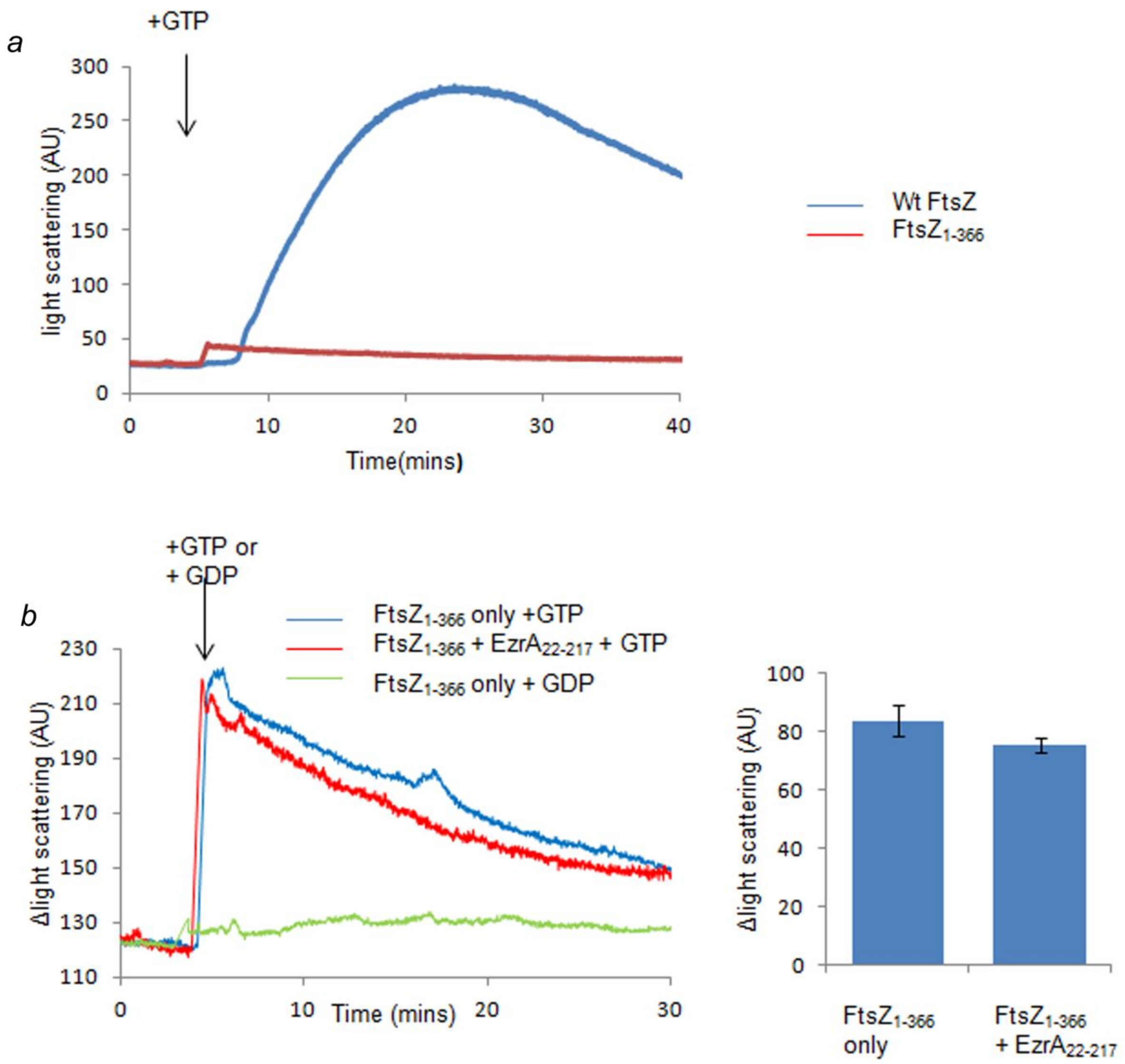

Supplementary Figure 10: EzrA spectrin repeats 1-2 require the FtsZ C-terminal tail to inhibit FtsZ polymerisation.

(**a**) Removal of the FtsZ C-terminal tail dramatically reduces FtsZ assembly, monitored by light scattering. There is a much reduced, but still significant, increase in light scattering on adding GTP to the FtsZ truncation mutant $FtsZ_{1–366.}$

(**b**) The GTP induced oligomerization of $FtsZ_{1–366}$ monitored by light scattering; the arrow corresponds to the time of GTP addition to 1 mM final concentration (left panel). *Bs*$FtsZ_{1–366}$ is present at 12.5 µM and *Bs*$EzrA_{22–217}$ at 25 µM. GDP addition does not affect scattering (data not shown). Average GTP-induced increase in light scattering for $FtsZ_{1–366}$ in the presence and absence of $EzrA_{22–217}$ (right panel); error bars represent the standard deviation from three independent measurements.

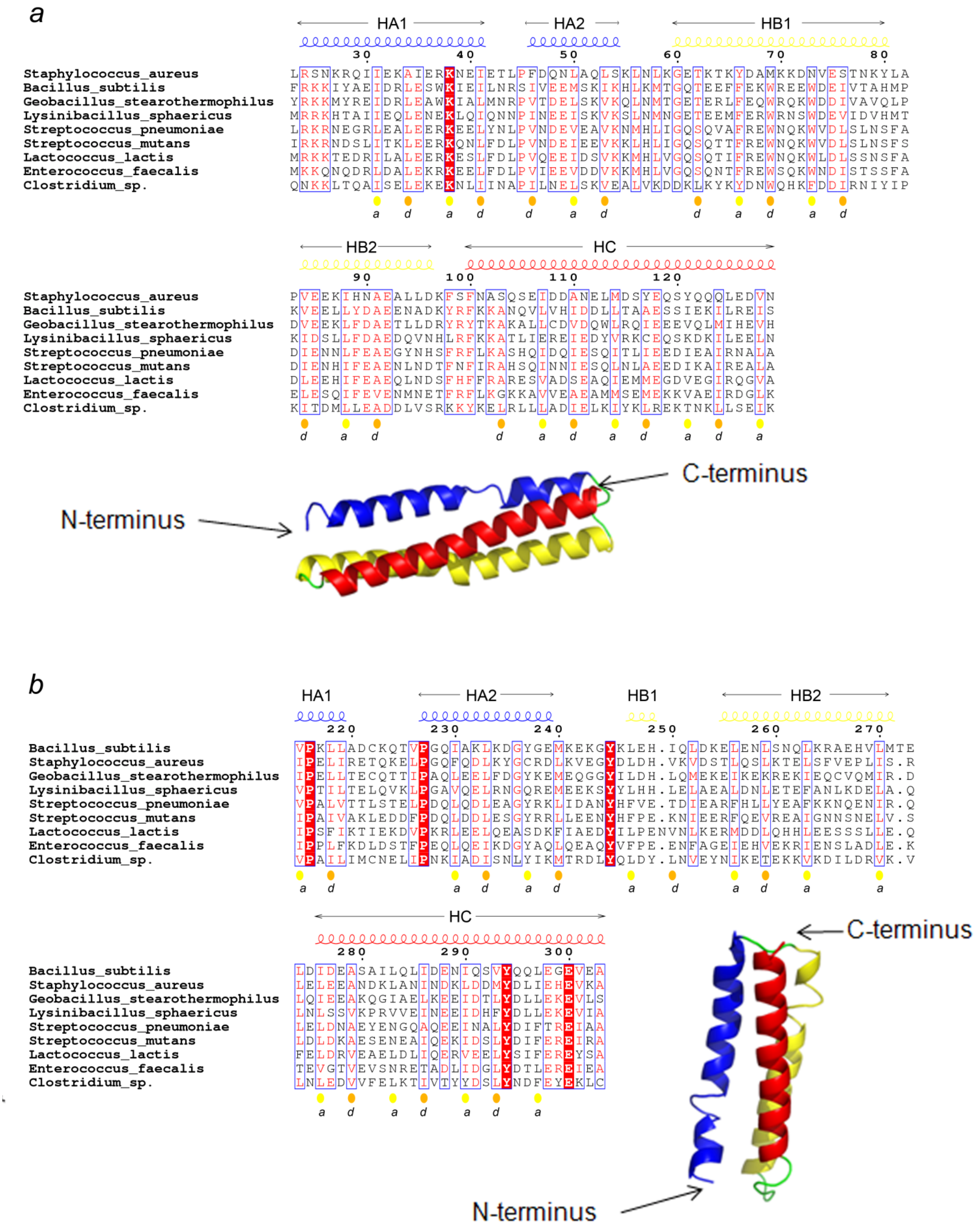

Supplementary Figure 11: Sequence alignment of EzrA paralogues in spectrin repeat units 1 and 3.

The alignment in panel (**a**) corresponds to the region encompassing the first spectrin repeat unit, the alignment in panel (**b**) encompasses repeat 3. The secondary structure elements at the top correspond to those from the *Sa*EzrA$_{24–214}$ structure in panel (**a**), and to those from residues 215–303 of the *Bs*EzrA$_{22-562}$ structure in panel (**b**). In both cases, the first helix in the repeat unit (Helix A) is coloured blue, the second orange (Helix B), the third red (Helix C); note that helices A and B are split into two component halves (i.e. HA1, HA2) due, in the case of repeat 1, to a proline residue in the centre of each helix that disrupts the helical geometry. There is also an absolutely conserved proline in helix A in repeat 3 (Pro227 in *B. subtilis*). The heptad periodicity of conserved, buried hydrophobic amino acids (positions *a* and *d* in the heptad repeat) is depicted beneath the alignments. Two conserved polar residues, a lysine at position 38 and a serine/threonine at position 62 are assigned to the *a* and *d* positions of the heptad repeat motif as they are buried in the interface between helices in the *Sa*EzrA$_{24–214}$ structure. Positions where residues have a similarity of >70% are in red ink and highlighted with a blue box, absolutely conserved residues are in white on a red background. All sequence alignment figures were prepared using the ESPRIT[17] server (http://espript.ibcp.fr/ESPript/ESPript/).

Supplementary Table 1: Parameters from kinetic fits to SPR titrations for the *Bs*EzrA$_{216–562}$:FtsZ interaction

Analyte	Model	Kinetic constants	RI, R_{max} (RU)
*Bs*EzrA$_{216–562}$	1:1	k_a = 870 ± 6 M^{-1} s^{-1}<br>k_d = (133 ± 2) x 10^{-5} s^{-1}	0.6–12.6, 265 ± 1
*Bs*EzrA$_{216–562}$	bivalent analyte	k_a = 211 ± 3 M^{-1} s^{-1}<br>k_d = (15 ± 2) x 10^{-3} s^{-1}<br>k_{a2} = (109 ± 8) x 10^{-6} RU^{-1} s^{-1}<br>k_{d2} = (16 ± 1) x 10^{-4} s^{-1}	2.1–14.5, 574 ± 12

Supplementary Table 2: Primer sequences used for PCR amplification.

Name	Sequence 5' – 3'
EzrA22NdeI	GGCAGGCTACCATATGAGGAAAAAAATCTACGC
EzrA562XhoI	CATGGTCGTCTCGAGCTAAGCGGATATGTCAGC
EzrA1NdeI	CAGCAAGGGGGCTCCATATGGAGTTTGTC
EzrA218STOP5	GATGATGTGCCGAAGTAGTTAGCTGACTGTAAACAGACGGTGC
EzrA218STOP3	GCACCGTCTGTTTACAGTCAGCTAACTACTTCGGCACATCATC
EzrA216fNdeI	CATAGATCATATGCCGAAGCTGTTAGCTGAC
EzrAstartRNdeI	CAATGACAAACTCCATATGGCTGC
Z377STOP5	CTTCACAGCCGGCTGATTAGACGCTTGACATCCCGAC
Z377STOP3	GTCGGGATGTCAAGCGTCTAATCAGCCGGCTGTGAAG
T7ftsZBamHI	GGAGGAGGATCCATGTTGGAGTTCGAAACAAACATAGACG
T7ftsZXhoI	CAAAATCGAACTCGAGTTTGTCCTTTACATTAGC
EzrAC222A5	CGAAGCTGTTAGCTGACGCTAAACAGACGGTGCCCGGAC
EzrAC222A3	GTCCGGGCACCGTCTGTTTAGCGTCAGCTAACAGCTTCG
EzrAK135C5	CTTGTCACAAGTGAGGAATGTAGCCGTGAAGAGATTGAACAGGTG
EzrAK135C3	CACCTGTTCAATCTCTTCACGGCTACATTCCTCACTTGTGACAAG
EzrAFor	GACTCTAGAGGAGTTTGTCATTGGATTATTAATTG
EzrARev	CTTAGGTACCGCGGATATGTCAGCTTTG
EzrA22	AGTTCTAGATAGGAAAAAAATCTACGCCGAAATCGACCG
EzrA217r	ATGGTACCAACTTCGGCACATCATCTATGTATGACTGAAGC
EzrA216	AGTTCTAGATCCGAAGCTGTTAGCTGACTGTAAACAGACGGTGCCCG
EzrA409	AGTTCTAGATAGAAAAGAAGAGCTTCAGGCGAGGGAGACGC

Supplementary Materials and Methods

Expression plasmid construction

The genes encoding *Bs*EzrA$_{22–562}$ and *Bs*EzrA$_{1–562}$ were cloned between the *Nde*I and *Xho*I restriction sites of pET28a, coding for an N-terminal hexahistidine tag and a thrombin cleavage site immediately upstream of the initiation codon. Primer sequences used to amplify coding sequences from *B. subtilis* strain 168 with appropriate flanking restriction sites are listed in **Supplementary Table 2**. The plasmid for expressing the *Bs*EzrA$_{22–217}$ fragment was prepared by Quikchange site-directed mutagenesis, using the pET28a derivative harbouring *ezrA*$_{22–562}$ as the template, and by introducing a stop codon in place of Leu218 with primers EzrA218STOP5 and EzrA218STOP3. The plasmid for expressing *Bs*EzrA$_{216–562}$ was derived from a pET28 derivative containing the *ezrA*$_{1-562}$ gene; the entire plasmid was PCR amplified with primers EzrA216fNdeI and EzrAstartRNdeI to generate a fragment omitting the DNA sequence equivalent to amino acids 1-215 of EzrA. This PCR product was then digested with *Nde*I and circularized with T4 ligase. The *Bs*EzrA$_{22–562}$ double mutant K135C/C222A was prepared by two steps of Quikchange PCR using primers EzrAC222A5, EzrAC222A3, EzrAK135C5 and EzrAK135C3. The plasmid pJB2076 was derived from the T7 promoter vector pETMCSI[18], and encoded residues 24-214 of *Sa*EzrA that were truncated from *E. coli* codon optimized *S. aureus ezrA*$_{24–564}$ (DNA2.0, USA) with a C-terminal tag of sequence HGSSGNSHHHHHHQL (the GNS sequence is encoded by an *Eco*RI site) appended. To shorten the C-terminal tag to improve crystal quality, pJB2076 was linearized with *EcoR*I and the ends were filled-in by Klenow polymerase and re-ligated with T4 ligase. This resulted in plasmid pZX2157, which encodes *Sa*EzrA$_{24–214}$ with 6 extra residues, HGSSGN, at the C-terminus. The plasmids pBS58 and pCXZ for overexpressing FtsZ were a gift from David Adams (Newcastle University). The plasmid for expression of FtsZ$_{1–366}$ was prepared by Quikchange site directed mutagenesis using plasmid pCXZ as template, introducing a stop codon in place of Asp367 using primers Z367STOP5 and Z367STOP3. The plasmid for expressing FtsZ with an N-terminal T7 tag was produced by amplifying the *ftsZ* gene from *B. subtilis* 168 genomic DNA using primers T7ftsZBamHI and T7ftsZXhoI; the gene was inserted between the *Bam*HI and *Xho*I sites of pET28a. In all cases, the correct DNA sequence was verified by dideoxy sequencing.

Expression and purification of *Bs*EzrA proteins

Escherichia coli strain BL21(DE3), containing pET28a-derivatives, was used for expression of *Bs*EzrA fragments. Cells cultures were grown at 37°C to an OD_{600} of 0.6–1 in LB broth containing 50 µg mL^{-1} kanamycin. The production of recombinant protein was induced by the addition of 0.1 mM IPTG

followed by overnight incubation at 25°C. For expression of selenomethionine-labelled proteins, the methionine-auxotrophic host strain B834(DE3) was grown in M9 minimal medium supplemented with 25 µg mL^{-1} $FeSO_4$; 1 µg mL^{-1} each of niacinamide, pyroxidine, riboflavin and thiamine; 40 µg mL^{-1} of each of the natural amino acids, with methionine replaced by selenomethionine. The B834(DE3) cells were grown to an OD_{600} of 0.4–0.8; protein expression was then induced with 1 mM IPTG followed by overnight incubation at 16°C.

Cells were harvested by centrifugation at 3,000 *g* for 30 min, and the cell pellet was resuspended in 20–30 mL lysis buffer (50 mM Tris.HCl, pH 8.0, 300 mM NaCl, 10 mM imidazole) per litre of culture. Roche complete protease inhibitor cocktail was added to approximately 0.4 times the final concentration recommended by the manufacturer, in addition to 5 mg lysozyme and *ca.* 200 units of DNase I per litre of culture. Cells were lysed by sonication and the lysate clarified by centrifugation at 45,000 *g* for 20 min at 4°C. The supernatant was filtered through a 0.45 µm filter and then loaded onto a 5 mL Ni-NTA cartridge (Qiagen) at 1.5 mL min^{-1}. After washing with *ca.* 100 mL lysis buffer, hexahistidine-tagged protein was eluted with lysis buffer containing 250 mM imidazole.

The hexahistidine tag was removed by overnight treatment with thrombin at 4°C (typically 1 unit of thrombin per 0.3 µg recombinant protein); the protein was then concentrated in a centrifugal concentrator to <5 mL and loaded onto a Superdex 200 or a Superdex 75 HR16/60 (GE Healthcare) gel filtration column pre-equilibrated in 10 mM Tris.HCl, pH 8.0, 250 mM NaCl. The appropriate protein-containing fractions were pooled and dialysed first against 10 mM Tris.HCl, pH 8.0, 50 mM NaCl, and then against 2 mM Tris.HCl, pH 8.0, 10 mM NaCl. Proteins were subsequently concentrated by ultrafiltration to 10–30 mg mL^{-1}, snap-frozen in liquid nitrogen and stored at –80°C prior to use. Protein concentration was calculated from the absorbance at 280 nm using the theoretical extinction coefficient.

The EzrA mutants used for EDC crosslinking experiments were expressed and purified by Ni-NTA chromatography by the same protocol as above, except after loading the lysate on the column, the column was washed with >15 column volumes of 50 mM Tris.HCl, pH 8.0, 300 mM NaCl, 10 mM imidazole, then 10 column volumes of 50 mM sodium phosphate, pH 8.0, 300 mM NaCl, 10 mM imidazole, then eluted with the same buffer containing 250 mM imidazole. The eluate was then concentrated to <5 mL and loaded onto a Superdex 200 HR 16/60 column (GE Healthcare) pre-equilibrated in 10 mM sodium phosphate, pH 7.7, 250 mM NaCl. Relevant fractions were then pooled and dialyzed against 10 mM sodium phosphate, pH 7.5, 150 mM NaCl, then concentrated to >10 mg mL^{-1} in a centrifugal concentrator. Aliquots of concentrated protein were flash frozen in liquid nitrogen and stored at -80°C.

Expression and purification of *Sa*EzrA$_{24–214}$

E. coli BL21(DE3)/pLysS containing pZX2157 was grown in LB medium (4 L) at 37˚C to an A_{600} of 0.6 ODU, then IPTG was added to 0.5 mM; cells continued to grow at 30˚C for 3.5 h before being collected by centrifugation. Pellets were stored at –80˚C. To purify the protein, cell pellets were resuspended in 60 mL of 50 mM Tris.HCl, pH 7.6, 10% sucrose, 100 mM NaCl, 10 mM spermidine, 5 mM dithiothreitol, 2 mM EDTA containing one tablet of Roche complete protease inhibitor cocktail. Egg-white lysozyme was added to 0.2 mg mL^{-1} and the suspension was stirred at 4˚C for 1 h. The lysate was cleared by centrifugation at 40,000 *g* for 30 min. $(NH_4)_2SO_4$ was slowly added to the supernatant to 0.28 g mL^{-1} with stirring for 1 h. The precipitate was removed by centrifugation at 40,000 *g* for 30 min. $(NH_4)_2SO_4$ was further added to a final concentration of 0.4 g mL^{-1}. Precipitated proteins were collected by centrifugation and dissolved in 45 mL of buffer A (30 mM Tris.HCl, pH 7.6, 15% glycerol, 2 mM dithiothreitol and 1 mM EDTA) and dialysed twice against two changes of 1 L of buffer A. A portion (35 ml) was loaded at 1 mL min^{-1} onto a 70 mL column of Toyopearl DEAE-650M (Tosoh Bioscience) equilibrated with buffer A. The column was washed with 100 mL of buffer A, and proteins were eluted with a linear gradient of 0–300 mM NaCl (500 mL) in buffer A. The flow-through and low-salt fractions containing *Sa*EzrA$_{24-214}$ were combined, dialysed against 1 L of buffer B (20 mM Tris.HCl, pH 7.6, 15% glycerol, 2 mM dithiothreitol and 1 mM EDTA). The dialysate was purified in three portions with an 8 ml Mono Q column (GE Healthcare) equilibrated with buffer B at 1 mL min^{-1}. The column was washed with 48 mL of buffer B and proteins eluted with a linear gradient of 0–400 mM NaCl (160 mL). Fractions containing nearly pure *Sa*EzrA$_{24–214}$ were combined, dialysed twice against 1 L of 20 mM Tris.HCl, pH 7.6, 1 mM dithiothreitol, 0.5 mM EDTA. The protein was concentrated with a centrifugal concentrator to 22.4 mg ml^{-1} for crystallization.

Expression and purification of FtsZ

Untagged *B. subtilis* FtsZ was co-expressed with *E. coli* FtsQ, FtsA and FtsW using plasmids pCXZ and pBS58, as described previously[19], using *E. coli* BL21(DE3) as host strain. The cell pellet from a 2 L culture was re-suspended in *ca.* 40 mL FtsZ lysis buffer (50 mM MES.NaOH, pH 6.5, 20 mM NaCl, 0.05 mM 4-(2-aminoethyl) benzenesulphonyl fluoride. 1mM EDTA) supplemented with one Roche complete protease inhibitor cocktail tablet. After lysis by sonication, the lysate was centrifuged at 45,000 *g* for 20 min and the supernatant then filtered through a 0.45-μm filter. The supernatant was loaded onto a XK16 column packed with 10 mL of Q-Sepharose (GE Healthcare) that had been pre-

equilibrated in FtsZ lysis buffer. After washing with *ca.* 100 mL lysis buffer, bound proteins were eluted in a linear gradient of 20–500 mM NaCl over 180 mL. To pooled fractions from the Q-Sepharose column, a solution of 80% (w/v) $(NH_4)_2SO_4$ in 50 mM MES pH 6.5, 250 mM NaCl was added to a final concentration of 20% (w/v), stirred for 30 min at 4°C then centrifuged in a JA-25.5 rotor at 19,500 rpm for 30 min. This step was repeated by the addition of $(NH_4)_2SO_4$ to the supernatant to a final concentration of 30% (w/v), which was stirred at 4°C for 30 min before centrifugation as above. The supernatant was supplemented with $(NH_4)_2SO_4$ to 40% (w/v), stirred and centrifuged as above. The pellet was resuspended in 4 mL of 10 mM MES.NaOH pH 6.5, 200 mM NaCl and loaded onto a Superdex 75 XK16/600 gel filtration column pre-equilibrated in the same buffer. Pooled fractions were dialysed against 10 mM MES.NaOH pH 6.5, 20 mM NaCl, concentrated to 20 mg mL^{-1} in a centrifugal concentrator and frozen in aliquots in liquid nitrogen.

The T7 tagged FtsZ was expressed, lysed and purified by Ni-NTA chromatography following the same protocol as for the EzrA fragments; the N-terminal His-tag was removed by overnight incubation with thrombin at 4°C (1 unit per 0.2-0.3 mg FtsZ), dialysed against 1 L of 10 mM sodium phosphate, pH 7.5, 200 mM NaCl, and concentrated to 7 mg mL^{-1} in a centrifugal concentrator. Aliquots of concentrated protein were flash frozen in liquid nitrogen and stored at –80°C.

Crystallisation and structure determination of *Bs*EzrA$_{22–562}$

*Bs*EzrA$_{22–562}$ was subjected to sparse matrix crystallisation screening by vapour diffusion in 96 well MRC plates with protein at a concentration of 32 mg mL^{-1}. An initial crystallisation hit was obtained in condition E1 of the Molecular Dimensions JCSG^{+} screen; the crystal morphology was optimised to the final crystallisation conditions of 50 mM MES.NaOH, pH 6.7, 1 M tri-sodium citrate, 0.1 M $(NH_4)_2SO_4$, and 0.2 M non-detergent sulphobetaine-201. For data collection, crystals were grown in hanging drops containing 1 µL of protein and 1 µL reservoir solution. Crystals were harvested after a week of growth by transfer first to 50% reservoir solution and 50% cryoprotectant solution (reservoir solution supplemented with glycerol to 15% v/v), then to 100% of the same cryoprotectant solution. After approximately 15 s, the crystals were flash frozen in liquid nitrogen.

A total of 4,898 images were collected at the Diamond light source from a single crystal grown from selenomethionine enriched protein. The *Bs*EzrA$_{22–562}$ structure was solved by single wavelength anomalous dispersion from the anomalous differences in the diffraction data set, which had been collected at wavelength 0.9795 Å, just above the selenium *K* atomic absorption edge. The diffraction

data were indexed and integrated in XDS[20] and scaled in SCALA[21]. The heavy atom substructure and initial phasing was determined using the SHELX[22] suite of programmes. Eight well-defined (occupancy >0.75) selenomethionine atoms were identified by the SHELXD[22] analysis of anomalous differences, which together with the absence of non-crystallographic symmetry, was consistent with a single EzrA molecule in the asymmetric unit. SHELXE[22] and PHASER[23] were used for subsequent density modification using the calculated crystallographic solvent content.

The atomic model was built by manually docking α-helices into the electron density in COOT[24]. The amino acid sequence was assigned by matching the positions of selenium atoms to methionine sulphur-atom positions in the protein sequence. The CCP4[25] program SLOOP[26] was used to find loops of the appropriate size which best fit the electron density map. The geometry of the loop-helix junctions was corrected using RAPPER[27], and PHENIX.REFINE[28] was used for refinement of the model. Summaries of the data collection and model refinement statistics are provided in **Table 1**. Disordered regions of the $EzrA_{22–562}$ structure were predicted from the amino acid sequence using the DisEMBL1.5 intrinsic protein disorder prediction server[29], http://dis.embl.de/. EzrA triple-helical bundles were structurally superimposed on spectrin-repeat containing proteins using secondary structure matching in COOT[24], and the PDBe MSDFold[30] structural alignment webserver with the ‘match connectivity’ option disabled. Angles between adjacent triple-helix bundle repeats in the EzrA and spectrin structures were determined using CHIMERA[31] to measure the angle between the principal axes of each repeat unit; each principal axis was determined from the centre of mass between each of the three helices in the repeat unit.

Crystallisation and structure determination of *Sa*EzrA$_{24–214}$

*Sa*EzrA$_{24–214}$ was also crystallised by sparse matrix crystallization screening using the sitting-drop vapour diffusion method and the JSCG+ screen (Qiagen) and 96-well plates. Initial crystals were grown by mixing equal volumes of protein and crystallization reagents (total volume 2 μL) prior to equilibration at 8°C. Several hits were obtained, the best of which grew from 70% (v/v) MPD, 0.1 M HEPES.NaOH, pH 7.5. The crystals subsequently used for X-ray data collection were grown using by hanging-drop vapour-diffusion in 24-well Linbro plates at 4°C with crystals grown from 2 μL protein at 15 mg mL^{-1} mixed with 2 μL of a reservoir solution of 34 % MPD, 0.1 M Tris.HCl, pH 7.5. Crystals were harvested after one week of growth, cryo-protected in reservoir solution and mounted in rayon fibre loops (Hampton Research) before plunging directly into a bath of liquid nitrogen.

X-ray diffraction data were initially collected from similarly grown crystals of C-terminally His_6-tagged *Sa*EzrA$_{24–214}$ at the Australian Synchrotron, but the data were anisotropic and incomplete at 2.35 Å.

Crystal quality was improved by use of the untagged protein, from which diffraction data were collected on beamline I04 at the Diamond synchrotron light source at a wavelength of 0.979Å. The data were indexed and integrated in XDS[20] and scaled in AIMLESS[32]. The structure was solved by PHASER[23] using domains 1 (residues 36 to 128 and 2 (residues 129 to 211) from the structure of *Bs*EzrA$_{22–562}$, the sidechains of which were modified by CHAINSAW[33] to remove non-conserved side-chains. The resulting model was subjected to iterative rounds of refinement in REFMAC[34] and manual rebuilding in COOT[24]. Summaries of the data collection and model refinement statistics are provided in **Table 1**.

EDC crosslinking

The relevant EzrA fragment (5 µM) was mixed with 10 µM of T7-tagged FtsZ in 10 mM sodium phosphate, pH 7.5 150 mM NaCl; for initial experiments reaction volumes of 20-40 µL were used. EDC was added to a concentration of 20 mM and the reaction then incubated at 22°C for 15 min, before adding 1M Tris.HCl, pH 8.0 (1/10th of the total reaction volume) to quench the reaction. To map the crosslink site on EzrA by NTCB cleavage, 1.2 mL crosslinking reactions were used; after quenching, the sample was diluted to 6 mL with 10 mM sodium phosphate, pH 8.0, 150 mM NaCl, 10 mM imidazole (buffer P1). A 200 µL bed volume of His select Ni-NTA slurry (Sigma Aldrich) was added. After incubation of the Ni-NTA slurry at 4°C with gentle agitation for >1 hour, the mixture was passed through an empty plastic chromatography column and the resin was washed with 5 mL buffer P1; with 8 mL 20 mM sodium phosphate, pH 8.0, 200 mM NaCl, 8M urea, 10 mM imidazole (P2); and 4 mL of 20 mM sodium phosphate, pH 8.0, 300 mM NaCl, 8 M urea, 10 mM imidazole (P3). Finally, His-tagged proteins were eluted from the resin with 1.2 mL of buffer P3 containing 250 mM imidazole and the eluate was concentrated to ~40 µL (1–2 A_{280} units) in a Vivaspin 500, 10 kDa cutoff concentrator. The Ni-NTA purified adduct (15 µL) was mixed with 15 µL of 8 M urea, 2 M glycine, pH 9.0, 10 mM NTCB, 2 mM dithiothreitol. The mixture was incubated for 4 h at 37°C, followed by SDS-PAGE and immunoblotting with the anti-T7 antibody.

Light scattering

Light scattering measurements used a Varian Cary Eclipse fluorimeter at 30°C with excitation and emission wavelengths set to 350 nm. All samples were in a buffer of 50 mM Tris.HCl, pH 7.3, 20 mM KCl, 3 mM magnesium acetate. Samples were pre-incubated for 5 min at 30°C, centrifuged in a microfuge and the supernatant transferred to a quartz microcuvette (10 mm path length, 3 mm window width). SDS-PAGE was used to verify that the protein concentration in the sample was not altered by

this pre-incubation and centrifugation step. After 5 min in the fluorimeter, GTP was added to a final concentration of 1 mM and light scattering recorded over >30 min. The GTP-induced change in light scattering in the presence of *Bs*EzrA proteins and *Bs*FtsZ was normalised relative to the average GTP-induced scattering change for *Bs*FtsZ alone, measured on the same day in the same apparatus using the same experimental procedures and buffers. The delay of 3-8 min before the scattering signal increased after addition of GTP, compared with an increase within seconds of GTP addition observed in previous studies[35,36] is attributed to the higher pH (pH 7.3) of the buffer used in our study. When we used the same buffer conditions (50 mM MES pH 6.5 50 mM KCl 2.5 mM $MgCl_2$ with 5 μM FtsZ and 1 mM GTP) as in previous reports[35,36], we also observed an increase in light scattering signal within seconds. However, the increase in pH, from 6.5 to 7.3, was necessary because *Bs*EzrA proteins aggregated in the pH 6.5 buffers typically used in this assay.

Surface plasmon resonance

All SPR experiments used a Biacore X100 instrument and a buffer of 10 mM HEPES.NaOH, pH 7.0, 150 mM KCl, 0.03 % Tween-20 with the chip surface at 25°C. For the titrations with *Bs*$EzrA_{216–562}$, a buffer of 10 mM MES.NaOH, pH 6.5, 150 mM KaCl, 0.03% Tween-20 was used. At the end of each injection the chip surfaces were regenerated by 15-20 s injection with 50 mM HEPES.NaOH, pH 7.0, 1 M NaCl, 0.5% dodecylmaltoside. *Bs*FtsZ was immobilised on Biacore CM5 chip surfaces at a concentration of 1 μM in 10 mM sodium acetate at pH 5.3. Chips were activated for amine coupling by standard protocols; after the desired level of protein had been attached to the surface, the activated carboxyl groups were quenched by injection of 1M ethanolamine, pH 8.0. In all cases a reference surface was prepared that was activated then directly quenched with ethanolamine. The binding data for *Bs*$EzrA_{22–217}$ were fit using the Biacore X-100 evaluation software; for the affinity fits the response units on the surface 4 s before the end of the injection were fit as a function of the concentration of the injected $EzrA_{22–217}$ fragment. Each SPR titration shown was performed independently at least twice. The relatively weak binding affinities reported by SPR are likely to overestimate the true affinity, since the physiological state, which is not replicated *in vitro*, requires EzrA to be embedded in the membrane to interact with polymers of FtsZ.

GTPase assay

The GTPase activity of FtsZ was measured using a continuous regenerative assay[16] that couples the production of GDP to the conversion of NADH to NAD^+. The same buffer conditions were used as in the light scattering assays, supplemented with 40 units mL^{-1} each of lactic dehydrogenase (Sigma Aldrich) and pyruvate kinase (Sigma Aldrich), 0.4 mM NADH, 1 mM phosphoenol pyruvate. Samples containing 12.5 µM *Bs*FtsZ and 25 µM of the relevant *Bs*EzrA fragment were pre-incubated at 30°C in Sterlin flat bottomed 96-well plates in a FLUOStar Optima plate reader (BMG Labtech) before GTP was added to 1 mM; the final volume of each reaction was 150 µL. The consumption of NADH was monitored by measuring absorbance at 340 nm; the absorbance was converted to NADH concentration based on the known extinction coefficient of NADH of 6220 M^{-1} cm^{-1}. The path length of the samples in the 96-well plate was determined by comparing UV absorbance measurements of the same sample in the plate and in a 1 cm path length cuvette in a standard UV spectrophotometer.

The malachite green assay measuring release of free phosphate again used the same conditions as used for the light scattering assays except the concentration of GTP was 2 mM, and *Bs*FtsZ and the relevant *Bs*EzrA fragment were at concentrations of 10 µM and 20 µM, respectively. The phosphate released after 20 min at 30°C was measured by mixing 8 µL samples of the reaction with 8 µL of 2 M HCl to stop the reaction, and 12 µL of this mixture were then mixed with 96 µL of a 3:2:1 mixture of water:0.08% malachite green:5.72% ammonium molybdate in 6 M HCl. 90 µL of this mixture were then mixed with 10 µL 32% sodium citrate and absorbance measured at 630 nm in a microplate reader. A standard curve was generated using phosphate ions in the concentration range 50 to 450 µM.

GTP binding

Fluorescence was measured at 22°C in a Varian Cary Eclipse fluorimeter using an excitation wavelength of 485 nm and emission wavelength of 535 nm with 5 nm excitation and emission slit widths. BODIPY GTP-γ-S (150 µL of 250 nM) in 50 mM HEPES.NaOH pH 7.0, 100 mM KCl, 5 mM $MgCl_2$ was preincubated for >5 min before adding 4–10 µL of a stock solution of either 8 µM FtsZ or 8 µM FtsZ with 16 µM $EzrA_{22–217}$ in the same buffer.

CD spectroscopy

CD spectra of *Bs*$EzrA_{131-217}$ were recorded at a protein concentration of 10 µM in 10 mM sodium phosphate buffer, pH 7.2, in 1 mm path length cuvettes in a JASCO J-810 spectropolarimeter with a PTC-4235 Peltier temperature controller. Wavelength scans were recorded at a scan rate of 10 nm min^{-1}

with a response time of 2 s, a data pitch of 0.2 nm and a bandwidth of 2 nm; the spectra represented are the average of 4 scans. For temperature scans, samples were heated at a rate of 1 °C min^{-1} with a response time of 8 s, a data pitch of 0.2°C and a bandwidth of 2 nm. The temperature scans was reversible, as spectra recorded before and after the scan were superimposable.

Analytical size exclusion chromatography

80 µL of 25 µM $EzrA_{22-562}$ in running buffer (50 mM Tris.HCl, pH 7.3, 20 mM KCl, 3 mM $Mg(OAc)_2$ or 10 mM Tris.HCl, pH 7.5, 150 mM NaCl) was injected onto a SuperdexHR10/300 column with a 100 µL injection loop, with the flow rate at 0.5 mL min^{-1}.

SEC-MALLS

200 µL samples at 0.5 mg mL^{-1} (*Bs*$EzrA_{216–562}$) or 1 mg mL^{-1} (*Bs*$EzrA_{22–217}$) were loaded onto a Superdex200 10/300 GL column (GE Healthcare) running at a flow rate of 0.75 ml min^{-1} in 10mM HEPES.NaOH, pH 7.0, 150 mM KCl (*Bs*$EzrA_{216–562}$) or 10 mM phosphate, pH 7.0, 100 mM NaCl (*Bs*$EzrA_{22–217}$). Samples eluting from the column passed through a Jasco UV-2077 detector, Wyatt DAWN Heleos II EOS 18-angle laser photometer with the 13th detector replaced with the QELS in-line dynamic light scattering detector. This was coupled to a Wyatt Optilab rEX refractive index detector and the molecular mass and concentrations of the resulting peaks were analysed using Astra 6.2.

Analytical ultracentrifugation sedimentation velocity

Sedimentation velocity (SV) experiments were carried out in a Beckman Coulter (Palo Alto, CA, USA) ProteomeLab XL-I analytical ultracentrifuge using interference and absorbance optics with an eight-place AnTi50 rotor. The AUC runs were carried out at 45,000 rpm at 20°C. The sample volume was 400 µL and sample concentrations ranged from 0.25 to 1.4 mg mL^{-1}. The protein's partial specific volume ($\bar{v}$) and the density and viscosity of the buffer (10 mM Tris.HCl, pH 7.0, 150 mM NaCl) at the experimental temperature were calculated using SEDNTERP[37]. Sedimentation velocity profiles were treated using the size-distribution $c(s)$ model implemented in SEDFIT[38]. Each peak on the distribution plot was integrated to obtain the weight-averaged values of the sedimentation coefficients under experimental conditions, which were subsequently converted to standard conditions ($s_{20,w}$), the S value in water at 20°C. The final values of sedimentation parameters for each sample were extrapolated to

zero concentration. Sedimentation coefficients were calculated from atomic co-ordinates using HYDROPRO[1] and SoMo[2].

Bacterial two-hybrid analyses

Two-hybrid plasmids were constructed by insertion of specific PCR fragments, amplified using the deoxyoligonucleotides listed in **Supplementary Table 2** (EzrAFor, EzrARev, EzrA22, EzrA216, EzrA217r, EzrA409), into pKT or p25N and pUT or pUTc using the *Kpn*I and *Xba*I sites incorporated into the amplifying deoxyoligonucleotides. This procedure allowed the orientated cloning and the correct fusion of *ezrA* or *ezrA* fragments with the respective fragment of the adenylate cyclase encoded on the plasmids as described in[39–41]. Appropriate combinations of pKT25 or p25N and pUT18 or pUT18c derivatives were simultaneously transformed into the adenylate cyclase-deficient *E. coli* strain BTH101. Following transformation, and having given sufficient time for the expression of the selection markers (25 μg mL^{-1} kanamycin and 100 μg mL^{-1} ampicillin), 8 μL aliquots of the transformation mixtures were spotted onto either minimal medium or nutrient agar selective plates containing 0.004% X-gal as an indirect reporter for interaction of the fusion proteins. The plates were then incubated at 30°C for 24 h prior to being photographed.

Supplementary References

1. Garcia de la Torre, J. Building hydrodynamic bead-shell models for rigid bioparticles of arbitrary shape. *Biophys. Chem*. **94**, 265–274 (2001).
2. Rai, N., Nöllmann, M., Spotorno, B., Tassara, G., Byron, O., Rocco, M. SOMO (SOlution MOdeler): differences between X-ray-and NMR-derived bead models suggest a role for side chain flexibility in protein hydrodynamics. *Structure* **13**, 723–734 (2005).
3. Jayasinghe, S., Hristova, K. & White, S. MPtopo: A database of membrane protein topology. *Protein Sci.* **10**, 455–458 (2001).
4. Nørholm, M., Shulga, Y., Aoki, S., Epand, R. & von Heijne, G. Flanking residues help determine whether a hydrophobic segment adopts a monotopic or bitopic topology in the endoplasmic reticulum membrane. *J. Biol. Chem.* **286**, 25284–25290 (2011).
5. Gupta, K., Selinsky, B.S., Kaub, C.J., Katz, A.K. & Loll, P.J. The 2.0 Å resolution crystal structure of prostaglandin H_2 synthase-1: Structural insights into an unusual peroxidase. *J. Mol. Biol.* **335**, 503–518(2004).
6. Borgon, R.A., Vonrhein, C., Bricogne, G., Bois, P.R. & Izard, T. Crystal structure of human vinculin. *Structure* **12**, 1189–1197 (2004).
7. Cansizoglu, A.E., Lee, B.J., Zhang, Z.C., Fontoura, B.M. & Chook, Y.M. Structure-based design of a pathway-specific nuclear import inhibitor. *Nat. Struct. Mol. Biol.* **14**, 452–454 (2004).
8. Grünwald, M. & Bono, F. Structure of Importin13-Ubc9 complex: nuclear import and release of a key regulator of sumoylation. *EMBO J.* **30**, 427–438 (2011).
9. Park, S.Y. Reconstruction of the chemotaxis receptor-kinase assembly. *Nat. Struct. Mol. Biol.* **13**, 400–407 (2006).
10. Masuda, M., Takeda, S., Sone, M., Ohki, T., Mori, H., Kamioka, Y., Mochizuki, N. Endophilin BAR domain drives membrane curvature by two newly identified structure-based mechanisms. *EMBO J*. **25,** 2889–2897 (2006).
11. Kusunoki, H., Minasov, G., Macdonald, R.I. & Mondragón, A. Independent movement, dimerization and stability of tandem repeats of chicken brain alpha-spectrin. *J. Mol. Biol*. **344**, 495–511 (2004).
12. Davis, L., Abdi, K., Machius, M., Brautigam, C., Tomchick, D.R., Bennett, V. & Michaely, P. Localization and structure of the ankyrin-binding site on β2-spectrin. *J. Biol. Chem*. **284**, 6982–6987 (2009).
13. Sreerema, N., Venyaminov, S.Y. & Woody, R.W. Estimation of the number of helical and strand segments in proteins using CD spectroscopy. *Protein Sci.* **8**, 370–380 (1999).
14. Whitmore, L. & Wallace, B.A. Protein secondary structure analyses from circular dichroism spectroscopy: Methods and reference databases. *Biopolymers* **89**, 392–400 (2008).
15. Kabsch, W. & Sander, C. Dictionary of protein secondary structure: pattern recognition of hydrogen-bonded and geometrical features. *Biopolymers* **22**, 2577-2637 (1983).
16. Ingerman, E. & Nunnari, J. A continuous, regenerative coupled GTPase assay for dynamin-related proteins. *Methods Enzymol.* **404**, 611–619 (2005).
17. Robert, X. & Gouet, P. Deciphering key features in protein structures with the new ENDscript server. *Nucleic. Acids Res*. **42**, W320-324 (2014).
18. Neylon, C., Brown, S.E., Kralicek, A.V., Miles, C.S., Love, C.A., Dixon, N.E. Interaction of the *Escherichia coli* replication terminator protein (Tus) with DNA: A model derived from DNA-binding studies of mutant proteins by surface plasmon resonance. *Biochemistry* **39**, 11989–11999 (2000).
19. Wang, X. & Lutkenhaus, J. The FtsZ protein of *Bacillus subtilis* is localized at the division site and has GTPase activity that is dependent upon FtsZ concentration. *Mol. Microbiol*. **9**, 435–442 (1993).
20. Kabsch, W. XDS. *Acta Crystallogr*. **D66**, 125–132 (2010).
21. Evans, P.R. Scaling and assessment of data quality. *Acta Crystallogr*. **D62**, 72–82 (2006).

22. Sheldrick, G.M. A short history of SHELX. *Acta Crystallogr*. **D64**, 112–122 (2008).
23. McCoy, A., Grosse-Kunstleve, R.W., Adams, P.D., Winn, M.D., Storoni, L.C. & Read, R.J. PHASER crystallographic software. *J. Appl. Crystallogr*. **40**, 658–674 (2007).
24. Emsley, P., Lohkamp, B., Scott, W. & Cowtan, K. Features and development of Coot. *Acta Crystallogr*. **D66**, 486–501 (2010).
25. Collaborative Computational Project Number 4. The CCP4 suite: programs for protein crystallography. *Acta Crystallogr*. **D50**, 760–763 (1994).
26. Cowtan, K. Completion of autobuilt protein models using a database of protein fragments. *Acta Crystallogr*. **D68**, 328–335 (2012).
27. Furnham, N., Doré, A.S., Chirgadze, D.Y., de Bakker, P.I., Depristo, M.A. & Blundell, T.L. Knowledge-based real-space explorations for low-resolution structure determination. *Structure* **14**, 1313–1320 (2006).
28. Adams, P.D. *et al.*PHENIX: a comprehensive Python-based system for macromolecular structure solution. *Acta Crystallogr*. **D66**, 213–221 (2010).
29. Linding, R., Jensen, L.J., Diella, F., Bork, P., Gibson, T.J. & Russell, R.B. Protein disorder prediction: implications for structural proteomics. *Structure* **11**, 1453–1459 (2003).
30. Krissinel, E. & Henrick, K. Secondary-structure matching (SSM), a new tool for fast protein structure alignment in three dimensions. *Acta Crystallogr*. **D60**, 2256–2268 (2004).
31. Pettersen, E. *et al.* UCSF Chimera--a visualization system for exploratory research and analysis. *J. Comp. Chem*. **25**, 1605–1612 (2004).
32. Evans, P.R. & Murshudov, G.N. How good are my data and what is the resolution? *Acta Crystallogr*. **D69**, 1204–1214 (2013).
33. Stein, N. CHAINSAW: a program for mutating pdb files used as templates in molecular replacement. *J. Appl. Crystallogr.* **41**, 641–643 (2008).
34. Murshudov, G.N., Vagin, A.A. & Dodson, E.J. Refinement of macromolecular structures by the maximum-likelihood method. *Acta Crystallogr*. **D53**, 240–255 (1997).
35. Buske, P. & Levin, P. A flexible C-terminal linker is required for proper FtsZ assembly *in vitro* and cytokinetic ring formation *in vivo*. *Mol. Microbiol.* **89**, 249-263 (2013).
36. de Oliveira, I.F. *et al.* Characterization of *ftsZ* mutations that render *Bacillus subtilis* resistant to MinC. *PLos One* **5**, e12048 (2012).
37. Laue, T.M., Shah, B.D., Ridgeway, T.M. & Pelletier, S. Computer-aided interpretation of analytical sedimentation data for proteins, In *Analytical Ultracentrifugation in Biochemistry and Polymer Science*, pp 90–125, Redwood Press Ltd, Melksham (1992).
38. Schuck, P. Sedimentation analysis of non-interacting and self-associating solutes using numerical solutions to the Lamm equation. *Biophys. J.* **75**, 1503–1512 (1998).
39. Claessen, D., Emmins, R., Hamoen, L.W., Daniel, R.A., Errington, J. & Edwards, D.H. Control of the cell elongation-division cycle by shuttling of PBP1 protein in *Bacillus subtilis*. *Mol. Microbiol*. **68**, 1029–1046 (2008).
40. Daniel, R.A., Noirot-Gros, M.F., Noirot, P. & Errington, J. Multiple interactions between the transmembrane division proteins of *Bacillus subtilis* and the role of FtsL instability in divisome assembly. *J. Bacteriol*. **188**, 7396–404 (2006).
41. Karimova, G., Pidoux, J., Ullmann, A. & Ladant, D. A bacterial two-hybrid system based on a reconstituted signal transduction pathway. *Proc. Natl. Acad. Sci. USA* **95**, 5752–5756 (1998).
